# Signatures of immune dysfunction in HIV and HCV infection share features with chronic inflammation in aging and persist after viral reduction or elimination

**DOI:** 10.1073/pnas.2022928118

**Published:** 2021-04-02

**Authors:** Cesar J. Lopez Angel, Edward A. Pham, Huixun Du, Francesco Vallania, Benjamin J. Fram, Kevin Perez, Thai Nguyen, Yael Rosenberg-Hasson, Aijaz Ahmed, Cornelia L. Dekker, Philip M. Grant, Purvesh Khatri, Holden T. Maecker, Jeffrey S. Glenn, Mark M. Davis, David Furman

**Affiliations:** ^a^Department of Microbiology and Immunology, Stanford University School of Medicine, Stanford, CA 94304;; ^b^Institute for Immunity, Transplantation and Infection, Stanford University School of Medicine, Stanford, CA 94304;; ^c^Division of Gastroenterology and Hepatology, Department of Medicine, Stanford University School of Medicine, Stanford, CA 94304;; ^d^Buck Institute for Research on Aging, Novato, CA 94947;; ^e^Davis School of Gerontology, University of Southern California, Los Angeles, CA 90007;; ^f^Stanford Center for Biomedical Informatics Research, Department of Medicine, Stanford University School of Medicine, Stanford, CA 94304;; ^g^Human Immune Monitoring Center, Stanford University School of Medicine, Stanford, CA 94304;; ^h^Division of Infectious Diseases, Department of Pediatrics, Stanford University School of Medicine, Stanford, CA 94304;; ^i^Division of Infectious Diseases, Department of Medicine, Stanford University School of Medicine, Stanford, CA 94304;; ^j^HHMI, Stanford University School of Medicine, Stanford, CA 94304

**Keywords:** aging, HIV, HCV, chronic inflammation, systems immunology

## Abstract

Chronic inflammation is thought to be a major cause of morbidity and mortality in aging, but whether similar mechanisms underlie dysfunction in infection-associated chronic inflammation is unclear. Here, we profiled the immune proteome, and cellular composition and signaling states in a cohort of aging individuals versus a set of HIV patients on long-term antiretroviral therapy therapy or hepatitis C virus (HCV) patients before and after sofosbuvir treatment. We found shared alterations in aging-associated and infection-associated chronic inflammation including T cell memory inflation, up-regulation of intracellular signaling pathways of inflammation, and diminished sensitivity to cytokines in lymphocytes and myeloid cells. In the HIV cohort, these dysregulations were evident despite viral suppression for over 10 y. Viral clearance in the HCV cohort partially restored cellular sensitivity to interferon-α, but many immune system alterations persisted for at least 1 y posttreatment. Our findings indicate that in the HIV and HCV cohorts, a broad remodeling and degradation of the immune system can persist for a year or more, even after the removal or drastic reduction of the pathogen load and that this shares some features of chronic inflammation in aging.

## Significance

Chronic inflammation contributes to morbidity and mortality in aging, but whether similar mechanisms underlie dysfunction in infection-associated chronic inflammation is unclear. Using a multicohort systems immunology approach, we identified signatures of immune dysfunction that are shared in aging and chronic viral infections, namely HIV and hepatitis C virus. We show that these shared dysfunctions persist despite viral clearance, and we describe the changes in functional coordination that occur during viral eradication. Finally, we highlight a partial restoration in interferon-α sensitivity across all major immune cell lineages as viral load drops. Our findings suggest a broad and persistent functional remodeling and deterioration of the human immune system despite removal of a chronic pathogenic burden that shares features of chronic inflammation in aging.

The immune dysfunction underlying persistent inflammatory states is a major contributor to global morbidity and mortality. Chronic infections, such as HIV and hepatitis C virus (HCV) infections, induce significant impairments in immune function over time including the progressive T cell dysfunction known as T cell exhaustion (1–5). Similarly, chronic inflammation in the absence of overt infection (sometimes referred to as sterile inflammation), is characterized by functional deterioration of immunity, exemplified by the immunosenescence of aging, and is associated with multimorbidity and mortality in older adults (6–14). Nonetheless, it remains unclear whether common molecular mechanisms underlie dysfunction in both aging-associated and infection-associated chronic inflammation, and if any of these functional alterations are reversible upon the removal of the inflammatory driver.

To address these questions, we analyzed three independent cohorts: 1) an aging cohort enriched for older individuals 61–90 y old; 2) a cohort of virologically suppressed, HIV-infected individuals on combination antiretroviral therapy (cART); and 3) a cohort of HCV-infected individuals prior to initiating treatment with the direct-acting antiviral (DAA) sofosbuvir—an NS5B nucleoside polymerase inhibitor which leads to viral clearance in a matter of weeks (15, 16). In each of these cohorts, we measured their immune cell composition and cellular signaling from peripheral blood mononuclear cells (PBMCs), and circulating cytokines, chemokines, and growth factors from serum samples, and utilized an established systems immunology analytical pipeline (12, 17–20) to characterize immune alterations associated with each of the three chronic inflammatory drivers. We monitored the HCV cohort throughout treatment as viremia dropped and up to a year after final treatment to determine which alterations persisted and which improved following viral clearance.

Previous studies have analyzed HCV patients treated with DAAs and found varying degrees of functional reconstitution of T cells (21, 22) and natural killer cells (NKs) (23, 24) following viral clearance. Studies focusing on NKs, for instance, have reported that HCV infection results in irreversible impacts on NK repertoire diversity (25), while others have shown an apparent return to normal regarding sensitivity in the effect of interferon (IFN)-α on signal transducer and activator of transcription 1 (STAT1) activation (26) after treatment. Consequently, some investigators (27, 28) have suggested that reestablishment of IFN homeostasis following chronic IFN stimulation may be crucial in achieving a sustained virologic response (SVR), defined as the absence of detectable HCV RNA 12 wk following the completion of treatment, which is highly predictive of long-term cure (29).

Using this multicohort systems approach, we identified overlapping dysregulations in immune composition and function in aging and infection-induced chronic inflammation, including an increase in memory T cells, increased intracellular inflammatory signaling, and a concurrent diminished sensitivity to cytokines in lymphocytes and myeloid cells. Although sensitivity to cytokine stimulation was partially restored in immune cells after viral eradication in the HCV cohort, global alterations of the immune system largely persisted. Our findings imply a profound and lasting effect on the human immune system after chronic infection.

## Results

### Shared Signatures in Aging-Associated and Infection-Associated Immune Dysfunction.

Three independent cohorts were analyzed in this study (*SI Appendix*, Tables S1–S3): 1) year two (2009) of the Stanford-Ellison longitudinal cohort on aging (12–14, 17–19, 30–32) consisting of 89 generally healthy and ambulatory individuals (*n* = 60 older individuals age 61–90, *n* = 29 young controls age 22–33); 2) an HIV cohort consisting of 69 individuals (*n* = 24 HIV-infected individuals age 26–78, *n* = 45 HIV-uninfected controls age 25–78); and 3) an HCV cohort consisting of 25 individuals (*n* = 14 HCV-infected individuals age 29–71, *n* = 11 HCV-uninfected controls age 18–74). For each cohort, we analyzed the abundance of major PBMC populations (*SI Appendix*, Fig. S1) including T cells, B cells, myeloid cells, and NK cells, each cell type’s STAT1, STAT3, and STAT5 baseline signaling states, and each cell type’s sensitivity to ex vivo cytokine stimulation. PBMCs from all cohorts were stimulated with type I and II IFNs, the aging and HIV cohorts were additionally stimulated with interleukin (IL)-2, IL-6, IL-7, IL-10, and IL-21, and the aging cohort was also stimulated via B cell receptor (BCR) cross-linking. We also measured a panel of 50 circulating cytokines, chemokines, and growth factors (17, 19) in each cohort (*SI Appendix*, Table S4). Because the HCV cohort served as a model of clearing a driver of chronic inflammation, we measured an additional 12 systemic proteins (*SI Appendix*, Table S4) and an additional nine immune signaling molecules to broaden our assessment (*SI Appendix*, Table S5). To determine the effect of age, HIV, and HCV status on the immune variables measured, we computed multiple regression analysis iteratively and adjusted for potential confounders of sex and cytomegalovirus (CMV) seropositivity in order to determine the effect of each driver of chronic inflammation on each feature. We derived a false discovery rate (FDR) for the effect of each explanatory variable on each feature, and found that 60, 25, and 130 features were significantly altered by aging, HIV, and HCV, respectively (FDR *Q* < 0.2) (*SI Appendix*, Fig. S2*A* and Tables S6–S8).

**Fig. 1.**
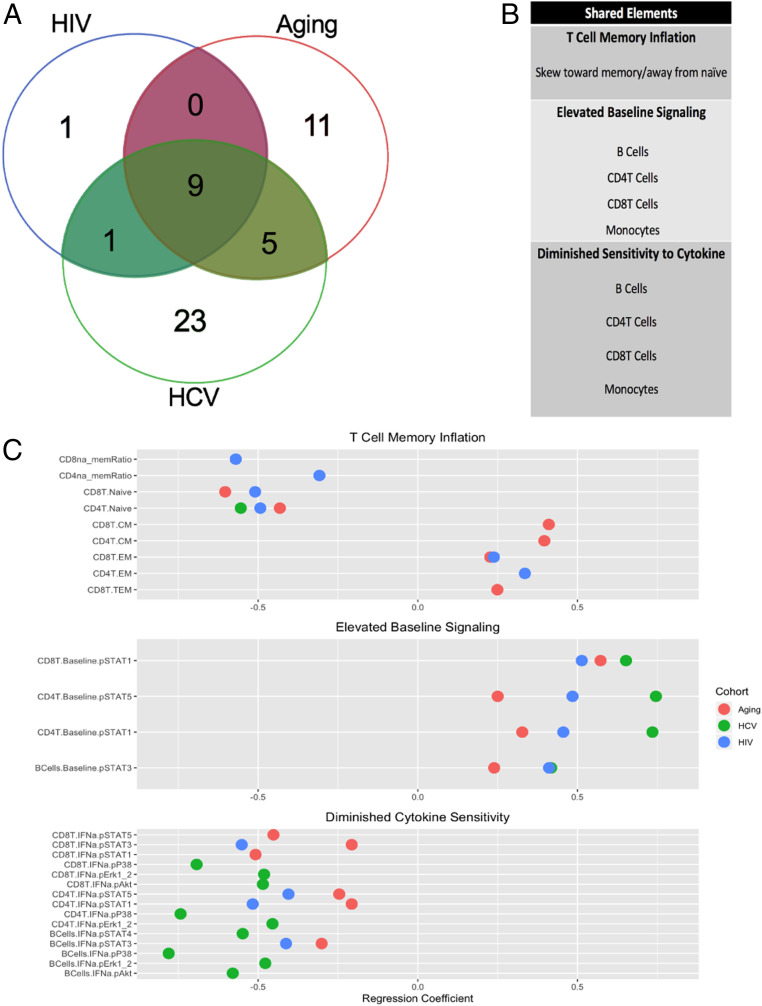
Shared immunological features of aging and infection-induced chronic inflammation. (*A*) Venn diagram summarizing the extent of functional overlap and differences between chronic inflammatory states induced by HIV, HCV, and aging. (*B*) Depicts the elements shared by all three cohorts. (*C*) Immunological features of chronic inflammation shared by each of the three cohorts are plotted with the effect size from the inflammatory driver (HIV, HCV, or Aging) on each feature represented by the regression coefficient. The magnitude of the regression coefficients are proportional to a larger effect size, with negative values indicating a dampening effect from the inflammatory driver and positive values representing a boosting effect.

To facilitate the interpretation of these results across cohorts, we classified each immune parameter into functional categories (*Materials and Methods*). Using this classification method, we identified 50 unique functional domains represented among the three cohorts (Fig. 1*A* and *SI Appendix*, Tables S6–S8), 9 of which were shared across the three cohorts (Fig. 1*A*), which we took as evidence of overlapping immune signatures associated with aging-associated and infection-associated chronic inflammation. In both age-associated and infection-associated immune dysfunction, an accumulation of memory and a commensurate dearth of naïve T cells, elevated baseline inflammatory signaling, and a diminished sensitivity to cytokine stimulation in lymphocytes and myeloid cells was observed (Fig. 1 *B* and *C* and *SI Appendix*, Fig. S2*B*).

With this method we identified additional shared features between pairs of cohorts. First, we found commonalities between the aging and HCV cohort including a decrease in γδ T cell frequency, a boosting of systemic IL-12p40 and tumor necrosis factor (TNF)-α cytokine levels, and enhancement of cytokine sensitivity in CD4^+^ and CD8^+^ T cells, primarily to IL-2 and IL-10 in aging and to IFN-γ in HCV (*SI Appendix*, Tables S6 and S7). Furthermore, both HCV and HIV infection imparted a negative effect on the frequency of CD28^+^ T cells (*SI Appendix*, Tables S7 and S8).

We additionally identified several immune parameters that were uniquely altered by each cohort. We found 23 functional perturbations uniquely associated with HCV, some of these perturbations were merely expansions of the functional domains shared by all three cohorts including elevated baseline inflammatory signaling and diminished sensitivity to cytokine stimulation in DCs, NKs, and γδ T cells (*SI Appendix*, Fig. S2 and Tables S6–S8). Other perturbations in this cohort were extensions of findings observed in both the HCV and aging cohort, such as enhanced sensitivity to IFN-γ in B cells, monocytes, NKs, DCs, and γδ T cells (*SI Appendix*, Tables S6 and S7). Unique functional consequences of HCV infection were attenuated baseline signaling via STAT4 in monocytes and via the mitogen-activated protein kinase (MAPK) pathway in B cells, monocytes, and NK cells. Systemic cytokine changes in HCV included decreased epidermal growth factor (EGF), elevated IL-1 receptor antagonist (IL-1RA), IFN-γ–induced protein (IP)-10, RANTES (regulated on activation, normal T cell expressed and secreted), Resistin, vascular cell adhesion molecule (VCAM)-1, vascular endothelia growth factor (VEGF), and VEGFD (*SI Appendix*, Table S7).

In the aging cohort, we observed an increased frequency of CD28^+^ T cells, NK cells, and monocytes, a diminished sensitivity to BCR stimulation, and an elevation in proinflammatory cytokines including IL-12p70, IL-18, IL-1β, IL-8, macrophage inflammatory protein (MIP)-1β, stem cell factor (SCF), and TNF-1β (*SI Appendix*, Table S6). As expected, a negative effect on CD4^+^ T cell frequency was observed only in the HIV cohort (*SI Appendix*, Table S8).

Overall, we defined shared signatures of immune dysfunction in both aging-associated and infection-associated chronic inflammation. Intrigued by the fact that the HIV-associated dysregulations were evident despite 8.3% of the cohort (*n* = 2/24) being virologically suppressed for more than a decade, we asked whether any of these shared perturbations could be improved with complete removal of the primary inflammatory driver.

### HCV Irreversibly Alters Global Immune Signaling Pathways.

To determine the extent to which these functional changes were reversible, we comprehensively characterized the HCV cohort over the course of viral eradication. To capture the most expeditious responses to the altered inflammatory milieu throughout DAA treatment, we designed a phospho-mass cytometry panel (*SI Appendix*, Table S5) to simultaneously interrogate the activity of 12 signaling hubs in seven PBMC lineages. This approach enabled systems-level profiling of four major immune signaling pathways: proliferation/survival/differentiation (P/S/D) represented by the phosphorylation of cAMP response element-binding protein (CREB), protein kinase B (Akt), and ribosomal S6 proteins; pathogen sensing/antiviral (PS/AV), represented by the expression of NFκB inhibitor alpha (IκBα) and phosphorylation of IFN regulatory factor 7 (IRF7); MAPK pathway, including extracellular signal-regulated kinase 1/2 (ERK1/2), MAPK-activated protein kinase 2 (MAPKAPK2), and p38 mitogen-activated protein kinases; and STAT signaling, including the transcription factors STAT1, STAT3, STAT4, and STAT5 (*SI Appendix*, Table S5). Each HCV-infected individual in the cohort was longitudinally profiled, and PBMC specimens were analyzed prior to sofosbuvir treatment, during the 12-wk regimen, and for up to 1 y after treatment (median = 9.5 mo, range = 5–13 mo).

**Fig. 2.**
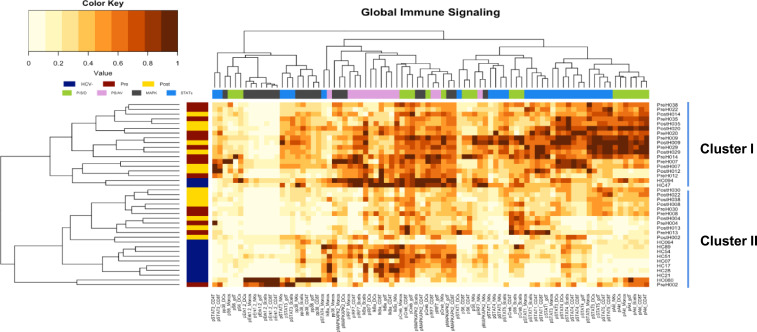
Global immune signaling separates HCV+ and HCV− patients. A heat map with hierarchical clustering of phosphorylated immune signaling molecules separates healthy/HC (cluster II) from HCV+ individuals (*P* = 0.03) with elevated inflammatory signaling evident in cluster I. Clustering of signaling parameters highlights the coordinated MAPK signaling (*P* = 4.4 × 10^−6^), STAT5-Akt axis signaling (*P* = 3.4 × 10^−8^), STAT signaling (*P* = 0.01), proliferation/survival/differentiation signaling (*P* = 0.01), and pathogen sensing/antiviral signaling (*P* = 1.9 × 10^−7^) across cell types. The optimal number of clusters was defined by gap statistic method, and enrichment *P* values were determined by hypergeometric test.

We began by comparing the global baseline immune signaling in all major immune cell subsets of HCV+ individuals pretreatment and posttreatment, and HCV-healthy controls (HC), through hierarchical clustering. This assessment revealed two clusters of individuals as the optimal cluster size defined by the gap statistic method (33) (Fig. 2 and *SI Appendix*, Fig. S3 *A* and *B*). Cluster I was enriched for HCV+ individuals and cluster II was enriched for HC (*P* < 0.05, by hypergeometric test). This suggested a separation between HCV+ and healthy individuals, but no clear distinction in global immune signaling between HCV infected individuals previral and postviral clearance.

We then applied the same approach to clustering of signaling parameters across individuals, and this revealed nine functional clusters (*SI Appendix*, Fig. S3 *C* and *D*). Analysis of these clusters highlighted a coordinated signaling across cell types within PS/AV (*P* = 1.9 × 10^−7^), MAPK (*P* = 4.4 × 10^−6^), STAT (*P* = 0.01), and P/S/D (*P* = 0.01) pathways, as well as a connection of STAT5 and Akt signaling (*P* = 3.4 × 10^−8^), an axis previously linked to immune cell proliferation and survival (34–36).

These results demonstrate that HCV infection induces gross dysregulation in immune cell signaling compared to HC via a global baseline elevation of phosphorylated hubs of multiple inflammatory signaling pathways across cell types and suggest only minimal changes in baseline signaling following viral clearance.

### Partial Restoration of Cytokine Sensitivity with HCV Viral Clearance.

**Fig. 3.**
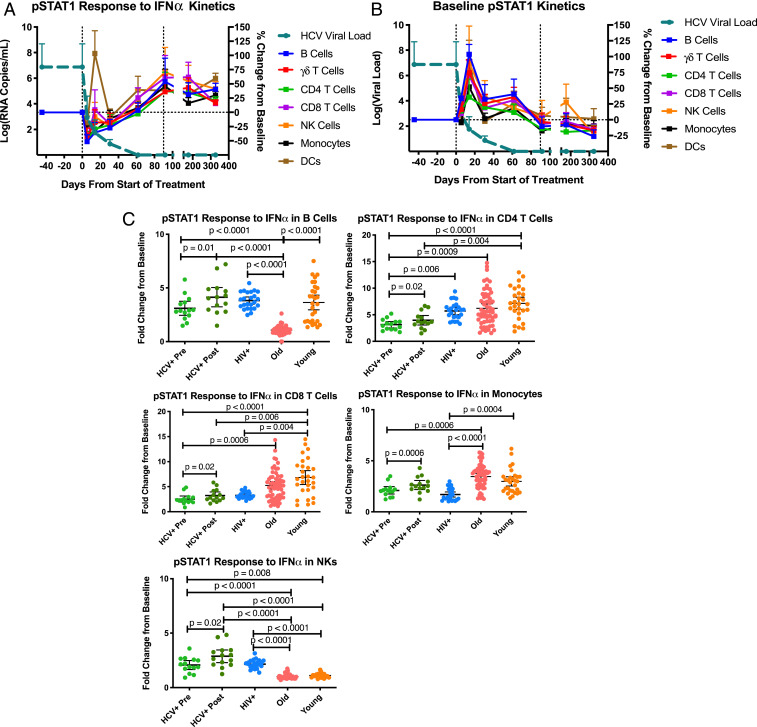
Restoration of pSTAT1 signaling in response to IFNα across immune cells following clearance or reduction of the viral stimulus. (*A*) Comparing the kinetics of viral load with the pSTAT1 response to IFNα stimulation in PBMCs of HCV+ individuals prior, during, and following sofosbuvir treatment. All paired, prepost comparisons had an FDR < 0.0001 by SAM. (*B*) The kinetics of HCV viral load and baseline pSTAT1 levels in PBMCs of HCV+ individuals prior, during, and following sofosbuvir treatment. The horizontal dashed line marks the baseline pSTAT1 response prior to treatment, while the vertical dashed lines mark the start and end of treatment, error bars are SE of mean for *A* and *B*. (*C*) pSTAT1 responses to IFNα stimulation in PBMCs across cohorts. Error bars represent 95% confidence intervals about the mean. Kruskal–Wallis tests performed comparing all groups to young or old group, and Wilcoxon matched-pairs signed rank tests performed comparing pre- and post-HCV+ groups.

Although our global clustering analysis did not reveal striking differences between treated and untreated HCV-infected individuals, we searched for significant changes pretreatment vs. posttreatment in all measured parameters individually with a two-class paired significance analysis of microarray (SAM) analysis. Of the 357 total measured features (*SI Appendix*, Fig. S2*A*), only 13 (4%) improved after sofosbuvir treatment (median = 9.5 mo posttreatment, FDR *Q* < 0.01) (*SI Appendix*, Table S9). These changes were most notable for their improvement of pSTAT1 signaling responses to IFN-α across all major cell lineages (Fig. 3*A* and *SI Appendix*, Table S9), but also included trends toward normalization of IκBα expression in monocytes, IFN-γ sensitivity in DCs, and levels of circulating plasminogen activator inhibitor (PAI)-1 an EGF (*SI Appendix*, Fig. S4 and Table S9).

To investigate the kinetics of this restoration of cytokine sensitivity, we analyzed the pSTAT1 responses to IFN-α in PBMCs of the HCV cohort during the course of sofosbuvir treatment and several months following the conclusion of treatment (Fig. 3*A*). The sensitivity to IFN-α in all measured PBMCs improved progressively during the course of DAA treatment (*P* < 0.05). In the first month of sofosbuvir administration, we observed a pulse in DC responses to IFN-α that peaked at the second week of treatment and coincided with a rapid reduction in viremia. Although significant improvements in CD8^+^ T cell and NK cell responses (*P* < 0.05) were also observed, this increase was relatively mild (Fig. 3*A*).

While we failed to detect a statistically significant change in baseline pSTAT1 levels, we followed the kinetics of unstimulated baseline pSTAT1 to contextualize the improved pSTAT1 responses to type I IFN (Fig. 3*B*). Intriguingly, we observed a similar pulse in all measured PBMCs concomitant with the rapid decrease in viral load, followed by a steady return of pSTAT1 level to baseline. Although we could not eliminate the possibility that sofosbuvir might have a direct effect on pSTAT levels in immune cells, such effect would be reasonably expected to persist continuously during exposure to the drug. In contrast, the observed spike and kinetics match models of viral clearance (37) and follow the viral load as it begins to fall.

To compare the observed improvement in IFN responses associated with viral clearance, we compared cell sensitivity to IFN-α across our three cohorts. We directly analyzed pSTAT1 signaling levels following ex vivo IFN-α stimulation of healthy, young individuals, i.e., the controls from the aging cohort, and used them as a reference to uncover the relative desensitization imposed by aging, HIV, and HCV (*Materials and Methods*). In this analysis, we again noted a desensitization to IFN-α in B cells due to aging (*P* < 0.0001), whereas CD4^+^ T cells (pretreatment *P* < 0.0001, posttreatment *P* = 0.004) and CD8^+^ T cells (pretreatment *P* < 0.0001, posttreatment *P* = 0.006) were desensitized by HCV infection relative to the controls (Fig. 3*C*). In contrast, CD8^+^ T cells (*P* = 0.004) and monocytes (*P* = 0.0004) were desensitized in HIV infection in their responses to IFN-α. Interestingly, IFN-α sensitivity was increased in NKs in both the HCV (pretreatment *P* = 0.008, posttreatment *P* < 0.0001) and HIV cohorts (*P* < 0.0001) relative to the controls (Fig. 3*C*).

Together these results confirm that the vast majority of immunological changes observed in HCV patients do not improve after clearance of the inflammatory trigger, but the partial restoration of the STAT1 immune signaling in response to IFN-α stimulation following viral clearance implies a relatively higher plasticity of this signaling axis.

### Immune Signaling Network Topology and Functional Coordination Are Altered with Antiviral Treatment.

**Fig. 4.**
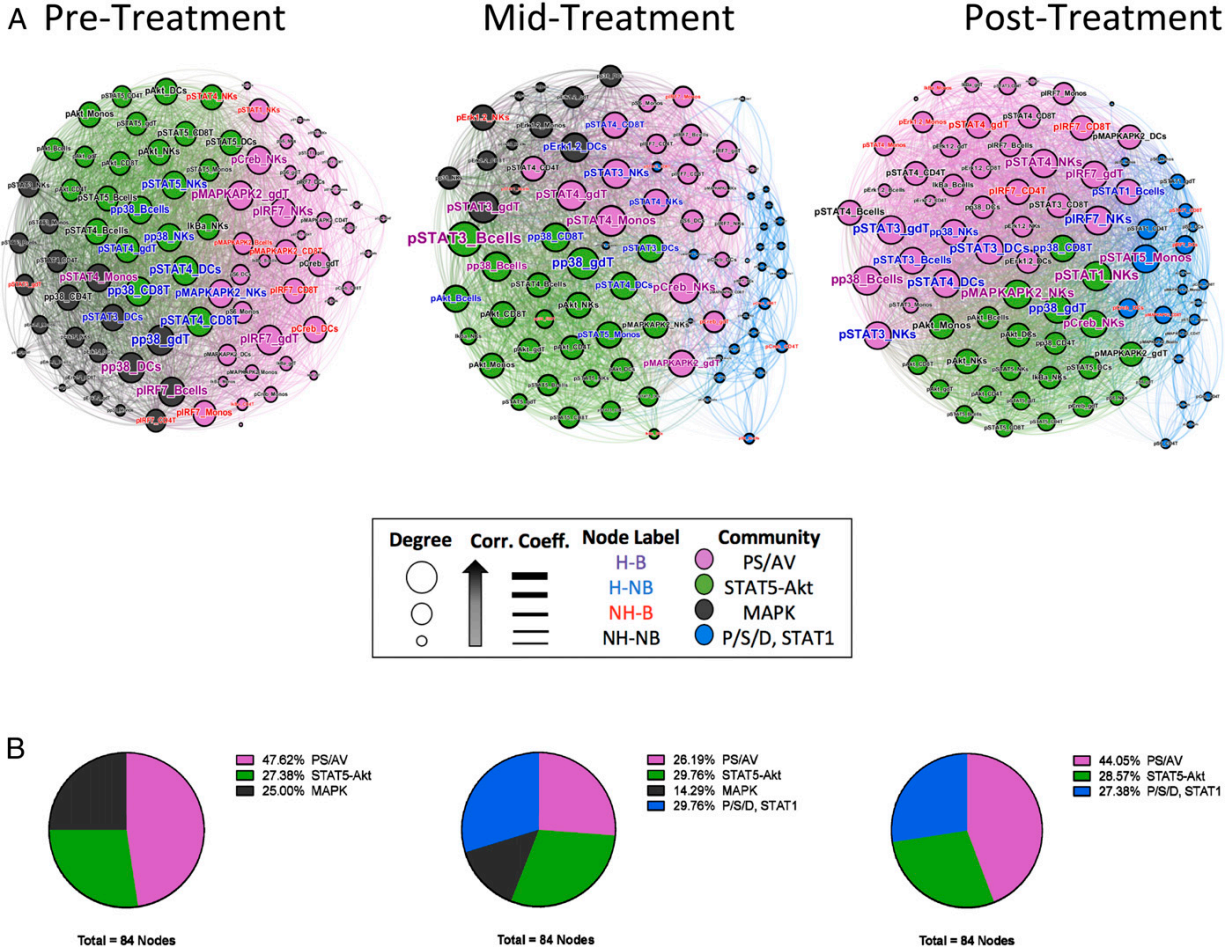
Immune signaling network topology and functional coordination are rewired with removal of the viral stimulus. Undirected correlation networks for HCV-infected individuals were generated by Force Atlas 2 algorithm layout. Spearman correlation matrices comprising 12 measured signaling parameters in seven major cell types were computed and visualized by plotting nodes of each immune parameter with weighted edges connecting nodes correlated by magnitude of correlation coefficient bigger than 0.5. Node diameters represented degree (connectivity). Edges were weighted by Spearman’s rank correlation coefficient. Edges were colored based on their source node. Community structure analysis (modularity analysis) was performed, and nodes and node labels were colored based on the feature legend. (*A*) The signaling network for HCV+ individuals (*n* = 14) prior to, midway through, and after sofosbuvir treatment. (*B*) Pie charts summarizing the percent number of each module (community) through the course of sofosbuvir treatment.

A strength of high-dimensional immune monitoring is the ability to simultaneously investigate fluctuations in individual parameters as well as system-level coordination. We sought to determine how the functional interconnectivity of immune parameters would respond to viral clearance. To investigate immune signaling network topology throughout the course of viral eradication, we generated undirected, weighted immune signaling correlation networks at the pretreatment, midtreatment, and posttreatment timepoints. Each network consisted of 84 nodes representing the 12 measured immune signaling parameters in each of the seven main PBMC cell types, with edges connecting nodes when the magnitude of their Spearman correlation coefficient is ≥ 0.5 (Fig. 4*A*). Each node is categorized as being either a network hub and a bottleneck (H-B), a hub-non bottleneck (H-NB), a nonhub bottleneck (NH-B), or a nonhub nonbottleneck (NH-NB) (38) (*SI Appendix*, Table S10 and *Materials and Methods*).

With this approach, we discovered that hubs and bottlenecks at each time-point were enriched for different immune signaling pathways. Pretreatment hubs were enriched for p38 and STAT4 signaling (*P* < 0.05), whereas midtreatment and posttreatment hubs were enriched for STAT3 and STAT4 (Fig. 4*A* and *SI Appendix*, Table S11). In pretreatment and posttreatment networks, the bottlenecks were enriched for IRF7 (*P* < 0.05), but no pathway was significantly enriched in midtreatment bottlenecks, reflecting the important role of bottlenecks in bridging multiple functional pathways (38) during the dynamic midtreatment state.

We studied changes in functional coordination during the course of sofosbuvir treatment by conducting modularity analysis (39) of each network to detect densely connected functional communities that are characteristic of human signaling networks (40) (Fig. 4 *A* and *B*). Modularity analysis revealed a total of four functional communities present in HCV+ individuals. These four modules were enriched either for PS/AV pathway activity, STAT5-Akt signaling previously linked to cellular proliferation and survival (34–36), MAPK pathway activity, or joint P/S/D and STAT1 activity, and colored in each network as pink, green, black, or blue nodes, respectively (Fig. 4 *A* and *B*). In the pretreatment network, only three communities were detected: The PS/AV (pink) module was the largest (47.6%), followed by STAT5-Akt (green, 27.4%), and the MAPK (black, 25%) module. However, in the midtreatment network the P/S/D, STAT1 (blue) community emerged (29.8%) partially replacing the PS/AV and MAPK communities and eventually completely replaced the MAPK population in the posttreatment network. This demonstrates that a signature of increased MAPK pathway activity in immune cells is characteristic of individuals with active HCV infection, and this shifts to a state of active STAT1 signaling in individuals who have cleared the virus, indicating that immune cell signaling closely tracks with disease states.

The strength of association and degree of nodes of the immune signaling network decreased during the treatment and returned to pretreatment levels following viral eradication (*SI Appendix*, Fig. S5 *A*, *B*, *E*, and *F*). Conversely, the betweenness centrality of network nodes, a measure of the number of shortest paths going through a given node (41, 42), and eccentricity, the maximum distance from a node to all other nodes and, thus, a measure of the functional influence of each node (42), increased during treatment and after viral clearance, reestablished to pretreatment levels (*SI Appendix*, Fig. S5 *C*, *D*, *G*, and *H*).

These results indicate that topological assessment of signaling networks can reveal dynamics of immune alterations that cannot be discovered using linear models, as measured from our cohort comparisons. Our findings highlight a dominant role of PS/AV signaling in patients infected with HCV, as well as a distinct transition from MAPK signaling to joint P/S/D and STAT1 signaling pathways that happened during the viral clearance process.

## Discussion

Systemic inflammation ensues as a response to chronic viral infections or as a maladaptive response with aging, and it has been associated with immune dysregulation and the development of multiple chronic conditions. To define similarities and dissimilarities of these inflammatory states in the remodeling of immune system components, we compared chronic inflammation in the context of aging, HIV, and HCV using a systems immunology approach. We found commonalities between aging-driven and infection-driven dysregulation including shifts from naïve to memory T cells, elevated baseline inflammatory signaling, and a diminished sensitivity to cytokine stimulation in lymphocytes and myeloid cells. These alterations were evident in the HIV cohort despite more than a decade of viral suppression in 8.3% of infected individuals and persisted for at least 1 y following viral clearance in the HCV cohort.

With recent pharmacological advances in DAAs, we now have therapeutics that completely eradicate HCV viremia very rapidly, which provides a rare opportunity to monitor the human immune system as it undergoes elimination of a chronic inflammatory stimulus. We relied on this model to investigate the degree of plasticity in the altered immune parameters observed in our cohorts and found that viral clearance mostly did not improve immune dysregulation. These findings suggest an imprinting of the human immune system by a chronic inflammatory insult. Nevertheless, partial restoration of pSTAT1 signaling in response to IFN-α indicates a degree of plasticity in this pathway, which makes it a candidate for potential therapeutic interventions targeting aging-related and chronic infection-related immune dysfunction.

Our findings on changes in immune cell composition are consistent with prior reports by our group (6, 12, 13, 17, 19) and validate findings observed in HIV (43, 44) and HCV cohorts (45). However, this study goes further by analyzing the immune system as a whole (46) and using the same standardized technological platforms across the three cohorts. Our findings reveal striking similarities in the early immune signaling events with elevation of phospho-protein content in multiple immune cell types from older individuals, and the HIV and HCV cohorts. This global rise in baseline phospho-proteins is concomitant with a reduced sensitivity to immune stimulations, likely due to the chronic exposure of circulating immune cells to a variety of inflammatory mediators in these cohorts. Despite major differences suggested between infection-driven and aging-driven inflammation (47), we find substantial overlap in the net effect of these different triggers of chronic inflammation (age-related versus infection-driven) in the immune system. Of a total of 50 immune system functional categories, 9 (18%) overlap between aging, HIV, and HCV. Of the 25 functional categories that significantly changed during aging, 9 (36%) overlap with HIV, and 14 (56%) overlap with HCV. Meanwhile, of the total of 11 functional categories that changed due to HIV, 10 (91%) are shared with HCV-infected individuals.

The analysis of each immune feature (cell subset, serum cytokines, cellular responses) shows that HCV infection has a more profound effect than that observed in HIV cases with 130/357 (36%) features being significantly different in HCV+ subjects vs. 25/128 (20%) in HIV+ patients compared to their healthy counterparts. This likely reflects the fact that the features assayed in our HCV cohort were more heavily skewed toward cell signaling events than in the HIV cohort. Alternatively, this may also be due to the fact that individuals in our HIV cohort were virologically suppressed by cART at the time of sample collection (range length of suppression = 13–145 mo), whereas our initial analysis of the HCV cohort was conducted on samples prior to DAA treatment. Aging also has a profound effect on immune system dysregulation similar to the ones observed in HCV patients with 60/166 (36%) being significantly altered in older adults. This indicates that the magnitude of the aging effect in the immune system is comparable to that observed in HCV patients. However, these effects did not fully overlap, suggesting that, contrary to previous reports (48, 49), chronic infectious diseases may not be an appropriate model for aging research, unless the overlap shown here is the focus.

Although HCV+ and HCV− individuals have clearly distinct immune signatures, we did not observe a pronounced shift following sofosbuvir treatment. It is possible that we were underpowered to detect subtle changes in our measured parameters, or that a similar analysis focusing exclusively on a younger population, in whom the immune system is inherently more plastic, would have yielded different results. Why most changes observed in HCV+ patients are not susceptible to modification with removal of the immune stimuli warrants further analysis.

Yet, our analysis reveals a clear rescue of pSTAT1 response to IFN-α stimulation in all major immune cell types, with a surge in DC sensitivity to type I IFN and in baseline pSTAT1 across cell types at precisely the time when mathematical models of HCV clearance predict mounting immunological pressure that affects viral load (37). The observed recovery of sensitivity to type I IFN could be explained by 1) a decrease in baseline (unstimulated) pSTAT1 levels while maximum pSTAT1 levels remain constant, yielding a greater fold change when stimulated with IFN-α ex vivo; 2) a recalibration of IFN-α signaling that allows for greater maximum pSTAT1 levels upon stimulation; or 3) a combination of these two scenarios. Because we failed to observe a statistically significant change in basal pSTAT1 signaling, we believe that the second scenario is more likely. While we did observe subtle changes in PAI-1 and EGF with treatment, and both cytokines can induce pSTAT1 signaling, we did not observe changes circulating cytokines that robustly initiate signaling via pSTAT1 such as type I or type II IFNs. While it is possible that there were significant changes in unmeasured cytokines that modulate pSTAT1 signaling, e.g., type III IFNs, we hypothesize that the restoration of sensitivity is likely to be, at least in part, cell intrinsic such as through changes in regulators of STAT1 phosphorylation.

One possible mechanism via which these signaling pathways are tuned following chronic HCV infection could be the epigenetic imprinting of chromatin in immune cells regulated by inflammatory mediators, with a more plastic regulation in the STAT1 system. Consistent with this hypothesis, our previous report (47) has shown that the interplay between epigenetics and environment can direct the expression of genes involved in inflammation into temporally distinct inflammatory responses (acute or chronic) with feed-forward loops of severe infections with systemic inflammation. This creates gene-specific silent facultative heterochromatin and active euchromatin concurring to gene function. It is therefore plausible that given the well-known essential role of STAT1 in the responses against infections and cancer, the function of the STAT1 signaling pathway is subject to rapid restoration because its regulators, including small ubiquitin-like modifier (SUMO), protein inhibitor of activated STAT (PIAS), and suppressor of cytokine signaling (SOCS) proteins are selectively reprogrammed by the profound immunological/inflammatory pressure caused by HCV and are epigenetic targets when the inflammatory trigger ceases.

Lastly, we observe a rearrangement of the network topology governing immune signaling during the course of treatment. Two primary functional modules representing PS/AV and STAT5-Akt activity were present in all networks, but we also observed a temporal shift away from MAPK signaling to a controlled STAT1-positive environment as viremia decreased. The presence of the MAPK community pretreatment is in line with previous studies demonstrating a concerted effort to maintain HCV-specific, T helper 1 (T_h_1) differentiation and IFN-γ production, beginning with antigen receptor signaling through p38 (i.e., MAPK) in lymphocytes (50–52). This anti-HCV differentiation is affected by STAT4 signaling (51, 53–57), which we also see in this community in CD4^+^ T cells and monocytes. The lack altogether of a dedicated MAPK functional module posttreatment suggests that the MAPK communities are at least partially maintained by HCV-specific antigen receptor signaling in lymphocytes (50–52, 58), as their abundance have been shown to increase during successful DAA treatment to reduce the antigenic burden (21, 59).

Intriguingly, STAT1 is the major anti-HCV immune signal transducer and is thus multimodally countered by HCV during chronic infection (60–62). The emergence of this community midtreatment is suggestive of the anti-HCV immune mobilization required to clear the infection, but its persistence after clearance, however, raises concern about the possibility of subsequent pathology secondary to prolonged inflammation (63).

In conclusion, we found common dysregulations of major immune signaling pathways with aging, HIV, and HCV and demonstrated that global alteration of immune system function persists after the removal of the inflammatory trigger with only partial restoration of STAT1 responses to IFN-α stimulation. It is unclear if other chronic viral infections with different life cycle dynamics, e.g., primarily latent with cycles of reactivation vs. primarily replicative, lytic infections, would result in different immune signaling imprinting. Unfortunately, we currently lack the pharmacological tools to directly answer this question directly in vivo for a majority of chronic infections. Given the potential implications in natural immunity and for vaccine development efforts, future studies are necessary to 1) determine whether this functional imprinting is inextricably related to the chronicity of the inflammatory stimulation, or whether relatively short-lived but vigorous inflammation, as in the setting of vaccination or SARS-CoV-2 infection (64, 65), also leaves this type of immunological footprint; and 2) to determine the effect of this signaling imprinting on downstream adaptive responses, as in the case of immunological imprinting in the context of influenza infection (66–69).

## Materials and Methods

### Phospho-Mass Cytometry Sample Processing and Staining.

Cryopreserved PBMCs stored at −180 °C were thawed in warm RPMI-1640 medium supplemented with 10% FBS, benzonase, and a penicillin streptomycin mixture (complete RPMI). Cells were transferred into serum-free RPMI medium containing 2 mM EDTA and benzonase, incubated with cisplatin for 1 min, and immediately quenched with four volumes of complete RPMI. One million cells per sample were then transferred into complete RPMI and rested for 30 min at 37 °C. Following resting, cells were stimulated with IFN-α (5,000 U/mL) or IFN-γ (50 ng/mL) and incubated for 15 min at 37 °C or left unstimulated. Cells were then fixed in phosphate-buffered saline (PBS) with 2% paraformaldehyde (PFA) at room temperature for 10 min, then washed 2× with CyFACS buffer and barcoded as previously described (70). Samples were combined for surface staining at room temperature for 1 h following barcoding. Subsequently, cells were washed and permeabilized in ice-cold MeOH at −80 °C overnight. The next day, cells were washed and incubated with the intracellular cytokine mixture at room temperature for 1 h. DNA stain was performed for 20 min with iridium (191/193) in PBS with 2% PFA at room temperature. Finally, cells were washed 2× with CyFACS buffer and 2× with MilliQ water before data acquisition on the CyTOF2 instrument. Data were debarcoded and manually analyzed on Cytobank (https://cytobank.org).

### Flow Cytometry Sample Processing and Staining.

For immunophenotyping, cells were thawed, washed twice with warm complete RPMI, resuspended at 1 × 10^7^ viable cells/mL, and 50 μL cells per well were stained for 45 min at room temperature with the following antibody mixture: CD3 AmCyan, CD4 PacificBlue, CD8 APCH7, CD28 APC, CD27PE, CD45RA PE-Cy5, CD19 Alexa Fluor 700, CD56PE, CD33 PE-Cy7, TCRgd APC (all from BD Biosciences). Cells were washed three times with FACS buffer (PBS supplemented with 2% FBS and 0.1% sodium azide) and resuspended in 200 μL of FACS buffer. One hundred thousand lymphocytes per sample were collected using DIVA 6.0 software on an LSRII flow cytometer (BD Biosciences). Data analysis was performed using FlowJo v9.3 by gating on live cells based on forward versus side scatter profiles, then on singlets using forward scatter area versus height, followed by cell subset-specific gating.

For phospho-flow, PBMCs were thawed in warm complete RPMI, washed twice, and resuspended at 0.5 × 10^6^ viable cells/mL, and 200 μL of cells were plated per well in 96-well deep-well plates. After resting for 1 h at 37 °C, cells were stimulated by adding 50 ng/mL of IFN-γ, IL-6, IL-7, IL-10, IL-2, or IL-21, or 5,000 U/mL of IFN-α, then incubated at 37 °C for 15 min. The PBMCs were then fixed with 2% PFA, permeabilized with ice-cold MeOH, and kept at −80 °C overnight. Each well was bar-coded using a combination of Pacific Orange and Alexa-750 dyes (Invitrogen) and pooled in tubes. The cells were washed with FACS buffer (PBS supplemented with 2% FBS and 0.1% sodium azide) and stained with the following antibodies (all from BD Biosciences): CD3 Pacific Blue, CD4 PerCP-Cy5.5, CD20 PerCp-Cy5.5, CD33 PE-Cy7, CD45RA Qdot 605, pSTAT-1 AlexaFluor488, pSTAT-3 AlexaFluor647, pSTAT-5 PE. The samples were then washed and resuspended in the FACS buffer. One hundred thousand cells per stimulation condition were collected and analyzed as above.

### Systemic Serum Protein Quantification.

This assay was performed in the Human Immune Monitoring Center at Stanford University. Serum samples were obtained by centrifugation of clotted blood and stored at −80 °C before cytokine level determination. Human 63-plex (HCV cohort) and 51-plex (aging and HIV cohorts) kits were purchased from eBiosciences/Affymetrix and used according to the manufacturer’s recommendations with modifications as described below. Briefly, beads (magnetic for HCV cohort, polystyrene for HIV and aging cohorts) were added to a 96-well plate and washed in a Biotek ELx405 washer. Serum samples were added to the plate containing the mixed antibody-linked beads and incubated at room temperature for 2 h (1 h for HCV cohort) followed by overnight incubation at 4 °C with shaking. Cold and room temperature incubation steps were performed on an orbital shaker at 500–600 rpm. Following overnight incubation, plates were washed in a Biotek ELx405 washer (HCV cohort) or vacuum filtered and washed twice with wash buffer (HIV and aging cohorts) and then biotinylated detection antibody was added for 2-h incubation (75 min for HCV cohort) at room temperature with shaking. Plates were washed again before streptavidin-PE was added. After incubation for 30–40 min at room temperature, plates were washed and the reading buffer was added to the wells. Each sample was measured in duplicate. Plates were read using a Luminex 200 instrument with a lower bound of 100 beads (50 for HCV cohort) per sample per cytokine.

All plates were run with a serum aliquot of an internal control and with AssayChex control beads (Radix Biosolutions) spiked to all wells. AssayChex quality-control beads (CHEX1, CHEX2, CHEX3, CHEX4) monitor instrument performance, application of detection antibody, application of fluorescent reporter, and nonspecific binding. Data were only considered for wells with nonspecific background fluorescence (CHEX4) within two SDs per plate, and for analytes meeting the bead count thresholds defined above. We then ensured that the mean fluorescence intensity for all remaining analytes were within the dynamic range of the assay and assigned values at the upper limit of quantification or lower limit of detection to the samples outside the dynamic range. Finally, we normalized each analyte to the internal control of each plate to minimize batch effects.

### Cross-Cohort Feature Selection and FDR Estimation.

To compare the effects of our three drivers of chronic inflammation across cohorts, we used linear regression models, as previously described (12), to identify immune measurements (features) that showed a statistically significant change in expression with age, HCV, or HIV status when adjusted for age, sex, and CMV seropositivity. Raw immune measurements were loaded into the R statistical software environment (https://www.r-project.org), standard score normalized within each cohort, and linear regression models were built. Mathematically, our models take the form:

$$Y_{ij}=\beta_{j}^{0}+\beta_{j}^{Age}\times Age_{i}+\beta_{j}^{Sex}\times Sex_{i}+\beta_{j}^{CMV}\times CMV_{i}+\beta_{j}^{HIV}\times HIV_{i}+\beta_{j}^{HCV}\times HCV_{i}+\epsilon_{ij},$$

where *Y*_*ij*_ is the intracohort standard score normalized measurement of immune parameter *j* in individual *i*. We applied the model to each data measurement in each cohort and computed *P* values of the β coefficients of age, sex, CMV, HIV, and HCV. We permuted the data measurement with respect to each of these variables 1,000 times, then recomputed the regression. For each permutation, we tested whether the absolute value of the permutation-derived β coefficients were greater or equal in magnitude to the absolute value of the true β coefficients. *P* values for each β coefficient were calculated as the ratio of the number of times the coefficients from the permuted regressions exceeded the betas from the true data regression over the total number of trials. To correct for multiple hypothesis testing, we considered the regression *P* values for all measurements of a single immune parameter simultaneously and calculated a q value (71) for each, and we considered any significant feature with FDR < 0.2.

Significant features from each cohort were then classified into functional domains as listed in *SI Appendix*, Tables S6–S8. For example, changes observed in memory CD4^+^ and CD8^+^ T cell frequencies were classified as “T cell memory skewing.” Changes observed in the expression of signaling proteins in unstimulated B cells, CD4^+^ or CD8^+^ T cells, or monocytes, were classified as “Baseline signaling in B cells,” “Baseline signaling in CD4^+^ T cells,” “Baseline signaling in CD8^+^ T cells,” and “Baseline signaling in monocytes,” respectively. Meanwhile, signaling protein expression in response to cytokine stimulations were binned by cell type into “Sensitivity to Cytokine in X cell type.”

Feature selection and regression coefficient plots from Fig. 1*C* and *SI Appendix*, Fig. S2 were generated in R using the ggplot2 library, while the Venn diagram of functional categories was rendered with http://bioinformatics.psb.ugent.be/webtools/Venn.

### Signaling Network Topology and Modularity Analysis.

Immune signaling correlation networks for HCV− and HCV+ individuals pretreatment, midtreatment, and posttreatment were generated by computing the Spearman correlation matrix in R for each of the 12 measured immune signaling parameters in each of the seven main PBCM cell types studied. We generated undirected, weighted immune signaling correlation networks from this matrix using the Force Atlas 2 algorithm layout on the open-source network analysis and visualization software Gephi, available at https://gephi.org. Network features and modularity analysis were exported from Gephi and curated manually to determine network hubs, i.e., the top 20% of nodes with the highest degree, and bottlenecks, i.e., within the top 20% of nodes with highest betweenness centrality, and to qualitatively classify each community from the modularity analysis.

Networks were visualized by plotting nodes of each immune parameter with weighted edges connecting nodes correlated by magnitude of correlation coefficient of at least 0.5. Nodes were colored based on community (module) to which they belong, and diameters were scaled by degree (connectivity). Edges are weighted by Spearman’s rank correlation coefficient and given the source node color. Hub-Nonbottleneck (H-NB) nodes are labeled in blue, Nonhub-Bottleneck (NH-B) nodes are labeled in red, Hub-Bottleneck (H-B) nodes are labeled in purple, and Nonhub-Nonbottleneck (NH-NB) nodes are labeled in black.

To validate the robustness of our networks, we generated null correlation matrices by permuting the median correlation values of each node in our observed networks 10,000 times and repeated this for each timepoint. We then computed the differences in median correlation values for each node in the observed data over time (∆_o_ pretreatment vs. midtreatment, pretreatment vs. posttreatment, and midtreatment vs. posttreatment) and repeated these calculations for the permuted null distributions (∆_n_). To determine *P* values for the differences in our observed networks over time and those of the null distribution, we computed the proportion of ∆_n_ that were at least as large as ∆_o_ for each node at each timepoint. We found that the observed differences in the majority of nodes (∼81%, *SI Appendix*, Table S12) were significantly different from changes in the null distributions and, therefore, concluded that our observed networks are robust.

## Supplementary Material

Supplementary File

## Data Availability

All study data are included in the article and/or *SI Appendix*.
