# Supplementary material for: Signatures of immune dysfunction in HIV and HCV infection share features with chronic inflammation in aging and persist after viral reduction or elimination
Source: Proc Natl Acad Sci U S A. 2021 Apr 2;118(14):e2022928118. doi: 10.1073/pnas.2022928118 (PMC8040665; doi:10.1073/pnas.2022928118)
Supplement: Supplementary File [file pnas.2022928118.sapp.pdf]

PNAS
www.pnas.org

Supplementary Information for

Signatures of immune dysfunction in HIV and HCV infection share features with chronic inflammation in aging and persist after viral reduction or elimination

Cesar J. Lopez Angel, Edward A. Pham, Huixun Du, Francesco Vallania, Benjamin J. Fram, Kevin Perez, Thai Nguyen, Yael Rosenberg-Hasson, Aijaz Ahmed, Cornelia L. Dekker, Philip M. Grant, Purvesh Khatri, Holden T. Maecker, Jeffrey S. Glenn, Mark M. Davis, and David Furman

Corresponding authors: Mark M. Davis, David Furman
Email: mmdavis@stanford.edu, furmand@stanford.edu

**This PDF file includes:**

SI Methods
Acknowledgements
Supplemental Figures S1-S5
Supplemental Tables S1-S12

## Supplementary Information (SI) Methods

*Human subjects and sample collection*

The Stanford-Ellison longitudinal cohort on aging was started in 2007 and continues under the direction of MMD and Dr. Scott Boyd. The cohort is part of the Stanford 1,000 Immunomes Project lead by MMD and DF. The data used here is from 2009 and was collected in that same year by the Stanford Vaccine Center directed by CLD, and analyzed by the Human Immune Monitoring Center at Stanford, directed by HTM. In 2009, the cohort consisted of 89 healthy individuals (N = 60 aged 61-90, and 29 aged ≤ 33 as controls). Subjects provided informed consent for participation in the study, and PBMCs and serum were drawn at time of recruitment. Exclusion criteria at time of enrollment were an active systemic or serious concurrent illness, a history of immunodeficiency, any known or suspected impairment of immunologic function, including clinically significant liver disease, diabetes mellitus treated with insulin, moderate to severe renal disease, blood pressure >150/95 mmHg at screening, chronic hepatitis B or C, recent or current use of immunosuppressive medication. None of the volunteers were recipients or donors of blood or blood products within the past 6 months and 6 weeks respectively, nor showed any signs of febrile illness on the day of blood draw.

The HCV cohort was recruited by AA and EAP from the Division of Gastroenterology and Hepatology in the Department of Medicine at Stanford Health Care in Palo Alto, CA, USA. PBMCs and serum samples were collected from 14 HCV-infected patients prior to initiating standard of care DAA therapy (Sofosbuvir and Ribavirin, Sofosbuvir and Ribavirin and IFN-α, or ledispavir/sofosbuvir) during treatment, and after treatment. Ten patients underwent at least one previous treatment with interferon, the other four were treatment naïve. Thirteen patients experienced SVR after twelve weeks of therapy, with one relapsing following the initial course. For uninfected controls, PBMCs were collected from 11 and serum from 10 healthy individuals from the Stanford Blood Center, Palo Alto, CA, USA.

The HIV cohort was recruited by PMG at the Division of Infectious Diseases in the Department of Medicine at Stanford Health Care in Palo Alto, CA, USA. This cohort comprises 24 virologically suppressed, HIV-infected individuals ages 26-78, and 45 uninfected healthy controls. All HIV-infected individuals had undetectable viral loads while on cART at the time of PBMC and serum specimen collection.

All serum specimens were aliquoted and stored at -80 °C and all PBMC specimens were aliquoted and stored at -180 °C. All human subjects provided written informed consent, and all study protocols were reviewed and approved by the Stanford University Institutional Review Board.

*CMV serology*

CMV serology for each study participant was determined using a commercially available ELISA kit (CMV IgG, Gold Standard Diagnostics, Davis, CA, USA) as per manufacturer's instructions. Briefly, sera stored at -80 °C were thawed to room temperature (20-25 °C) and diluted 1:51 in kit diluent. Diluted samples were added to wells coated with CMV antigen from strain AD169 and incubated at room temperature for 30 minutes. Wells were washed and drained, followed by the addition of goat anti-human IgG antibodies labeled with calf alkaline phosphatase, and incubated at room temperature for 30 minutes. Wells were washed again and drained, followed by the addition of p-nitrophenyl phosphate substrate, and incubation at room temperature for 30 minutes. After the addition of 0.5M trisodium phosphate stop solution, the absorbance of each well at 405 nm was read and results were analyzed using the manufacturer's instructions.

*Enrichment analysis*

Cluster enrichment analyses were performed by computing p-values for under- or over-enrichment based on the cumulative distribution function of the hypergeometric distribution using the hypergeometric p-value calculator available at https://systems.crump.ucla.edu/hypergeometric/. Mathematically, the hypergeometric probability is expressed as:

$$Pr(x; N, n, k) = \frac{\binom{k}{x}\binom{N-k}{n-x}}{\binom{N}{n}}$$

where $x$ is the number of successes within a sample of size $n$ drawn from a population of size $N$ containing $k$ total successes.

When samples are drawn with replacement, as in the case of node enrichment for network hubs and/or bottlenecks, the probability function follows a binomial distribution and is expressed as:

$$Pr(k; n, p) = \binom{n}{k} p^k (1-p)^{n-k}$$

where $k$ is the number of successes in $n$ trials, and $p$ is the probability of a success. In these cases p-values were calculated with https://stattrek.com/online-calculator/binomial.aspx.

*Hierarchical clustering analysis*

Raw immunological measurements were intra-cohort min-max features scaled in R, and hierarchical clustering and heatmaps were generated in R with the gplots library. The optimal number of clusters were defined by gap statistic (33) method. Briefly, this method clusters the input data, varying the number of clusters from $k$ = 1,…, $K$ and computes the total within-cluster dispersion $W_k$ for each $k$. Next, $B$ reference data sets are generated from a random uniform distribution, and each is clustered as above and within-cluster dispersions $W^*_{kb}$, $b$ = 1, …, $B$, $k$ = 1,…, $K$ are computed. The estimated gap statistic is then computed as follows:

$$Gap(k) = \left(\frac{1}{B}\right)\sum_{b=1}^{B} log\,(W^*_{kb}) - log\,(W_k)$$

and the standard deviation of the statistics $s_k$ is also computed. Finally, the number of clusters is chosen as the smallest value of $k$ such that the gap statistic is within one standard deviation of the gap at $k$+1: $Gap(k) \geq Gap(k+1) - s_{k+1}$. Gap statistics were computed on scaled data and plotted in R with the factoextra and NbClust libraries with 500 Monte Carlo bootstrap samples. Clusters were then determined by partitioning the clustering dendrograms at the highest level that yielded the optimal number of clusters $k$. Approximately unbiased p-values for clusters were computed by multiscale bootstrap resampling (10,000 bootstrap samples) using the pvclust R package, and clusters meeting the significance threshold of $\alpha$ = 0.05 were highlighted.

*Additional statistical analyses*

Two-class paired significance analysis of microarray (SAM) were performed with MultiExperiment Viewer available at http://mev.tm4.org/. FDR was determined by q-values generated by running the analysis with all 16,384 possible permutations. All additional statistical tests were performed with Graphpad Prism software v7.0d and v8.2.1 for Mac OS X (La Jolla, CA, USA).

*Data availability*

The data and R code used to generate the figures in this manuscript are available at https://github.com/CesarLopezAngel/AgingHCVHIV.

*Author Contributions*

CJLA, EAP, JSG, MMD, and DF conceptualized the study. CLD recruited and enrolled participants into the Stanford-Ellison longitudinal cohort on aging, and PMG recruited and enrolled the HIV cohort. Aging and HIV cytometry and cytokine data were collected and analyzed by the Human Immune Monitoring Center at Stanford led by HTM. AA and EAP recruited the HCV cohort. TN and BJF processed HCV specimens supervised by JSG and acquired HCV cytokine data together with YR-H. FV, BJF, CJLA, and YR-H analyzed cytokine data supervised by HTM and PK. CJLA designed the mass cytometry panel and acquired and analyzed mass cytometry data supervised by MMD, and performed network modularity analysis supervised by DF. CJLA, DF, and XD performed multi-cohort statistical integration and generated final figures with KP. CJLA, DF, XD, KP, and MMD wrote the manuscript with input from all authors.

**Acknowledgements**

We thank Dr. Bali Pulendran for critical review of the manuscript. We also thank Dr. Winston Haynes for his assistance with the modularity analysis. CJLA and EAP were supported by the Paul and Daisy Soros Fellowship for New Americans, the Howard Hughes Medical Institute (HHMI) Medical Research Fellows Program, and Stanford's Medical Scientist Training Programs at the time the research was conducted. Mass cytometry data was collected on an instrument in the Stanford Shared FACS Facility obtained using NIH S10 Shared Instrument Grant S10OD016318-01. This research was supported by NIH Grant U19 AI057229 to MMD and a Stanford Translational Research and Applied Medicine (TRAM) pilot grant to MMD and AA. The ClinicalTrials.gov number for the aging cohort is NCT 01827462.

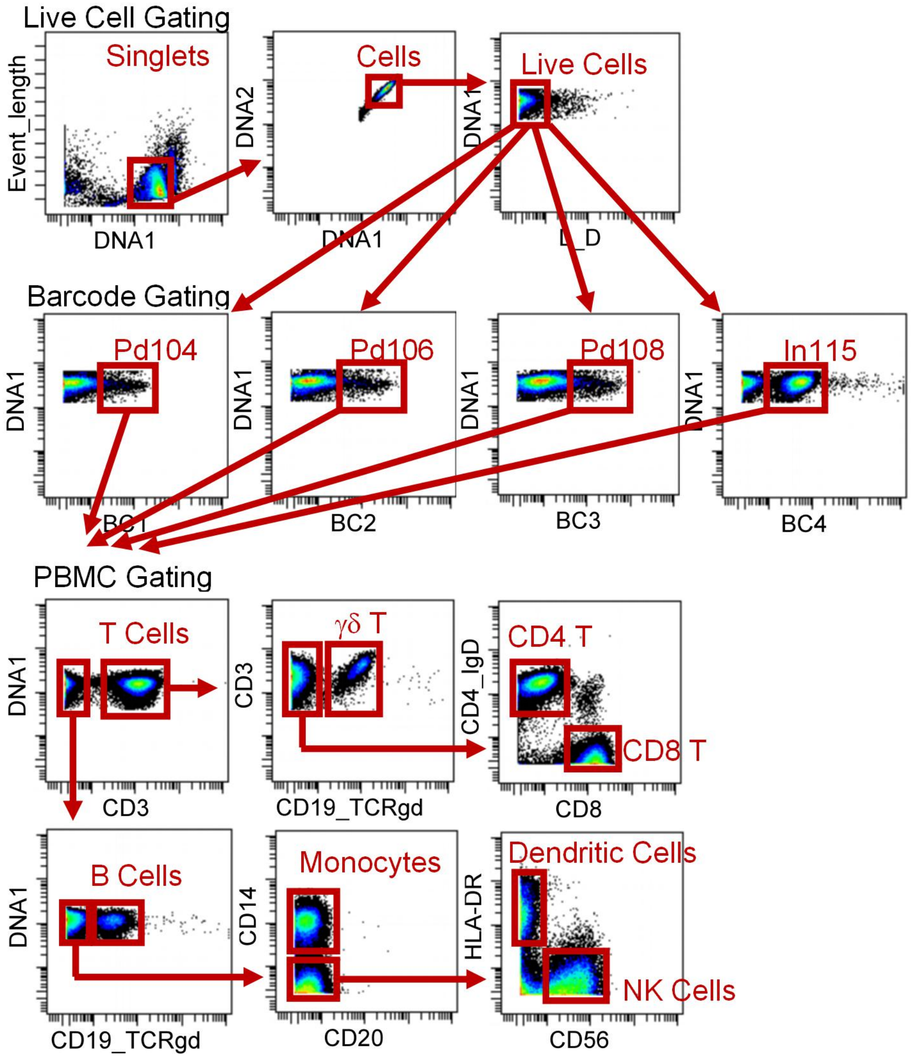


**Figure S1. Manual gating strategy for CyTOF.** Representative gating scheme to identify the seven major PBMC populations used for comprehensive analysis of cell frequencies and signaling states, namely $\gamma\delta$, CD4, and CD8 T cells, B Cells, Monocytes, Dendritic Cells, and NK Cells.

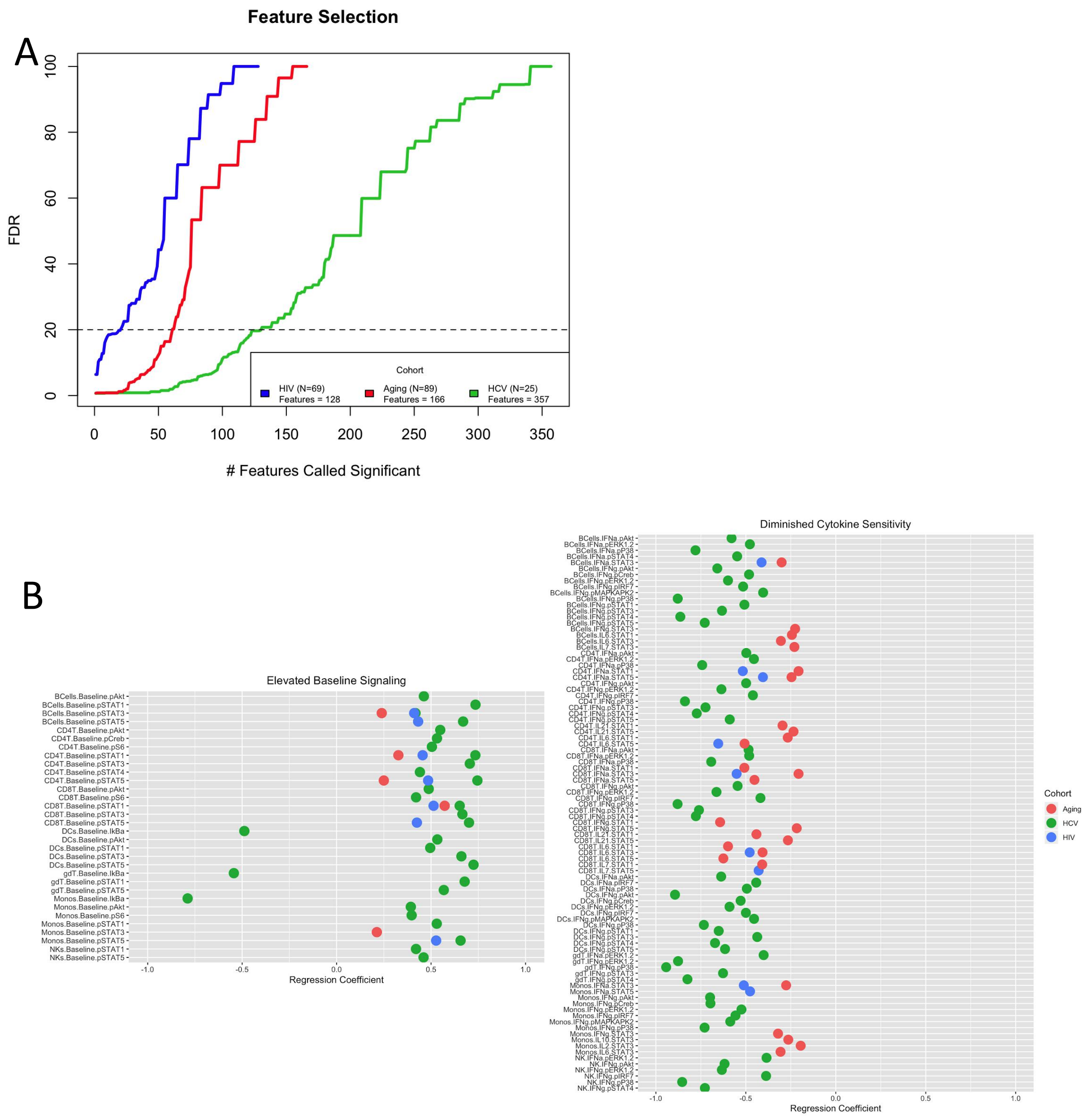


**Figure S2. Shared immunological features of aging and infection-induced chronic inflammation.** A) The number of features meeting the significance threshold of FDR < 0.2 for each cohort is shown. The effect of HIV, aging, and HCV were determined by iterative linear regressions adjusting for confounders of sex and CMV seropositivity, and FDRs computed following 1,000 permutations.

B) The complete set of immunological features that belong to the shared functional categories between HIV, aging, and HCV cohorts.

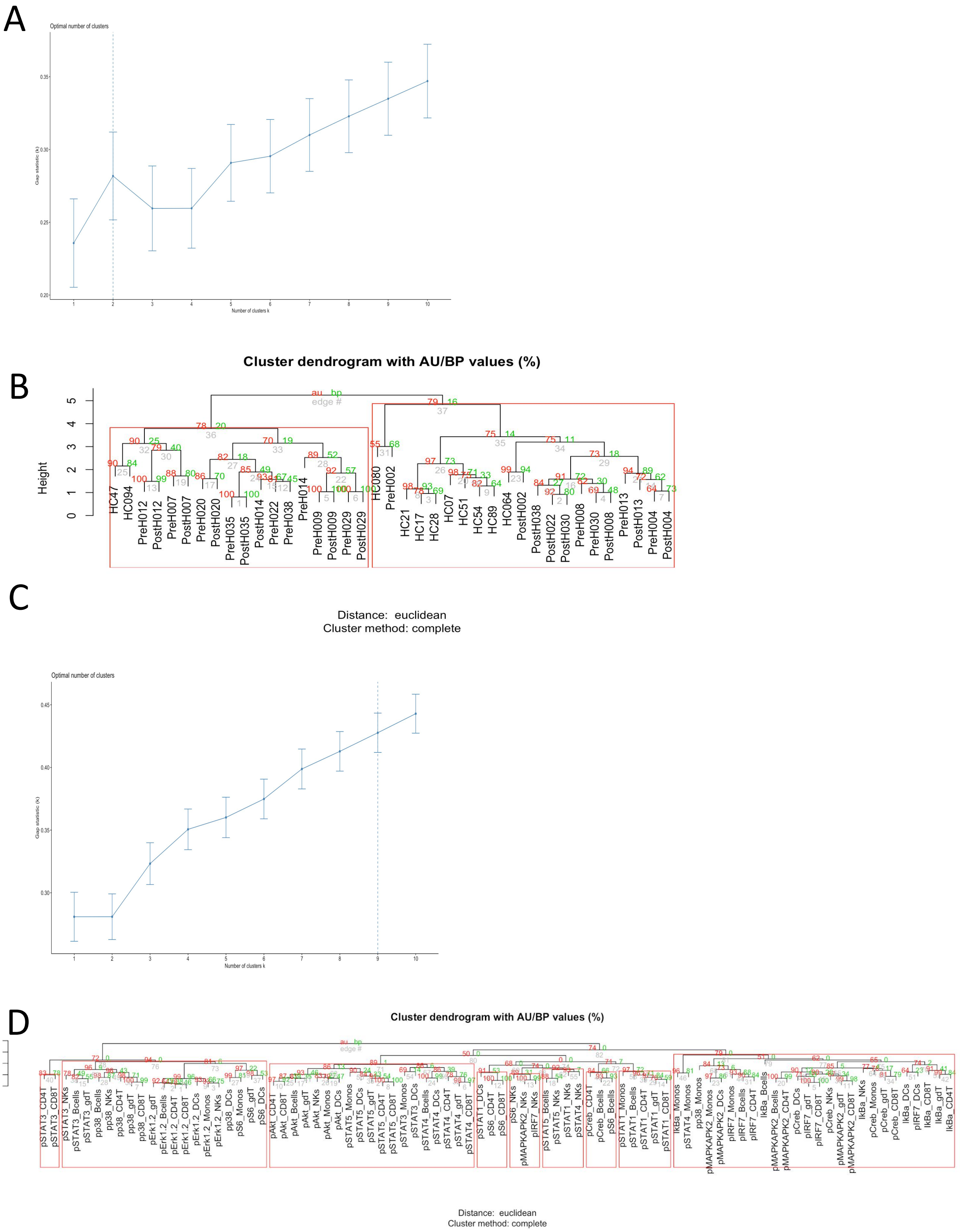


**Figure S3. Hierarchical clustering of global immune signaling separates HCV+ from HCV- individuals and defines functional clusters.** A) Gap statistics plotted as a function of number of clusters identifies two optimal clusters within the HCV study participants. B) Cluster dendrogram of HCV study participants demonstrates highest two-cluster partition. AU p-values (red) and bp values (green) for each branch are shown. C) Gap statistics plotted as in A demonstrating nine optimal functional signaling clusters. D) Cluster dendrogram of immune signaling parameters in major PBMC cell types demonstrates the highest nine-cluster

partition with AU p-values and bp values plotted as in B.

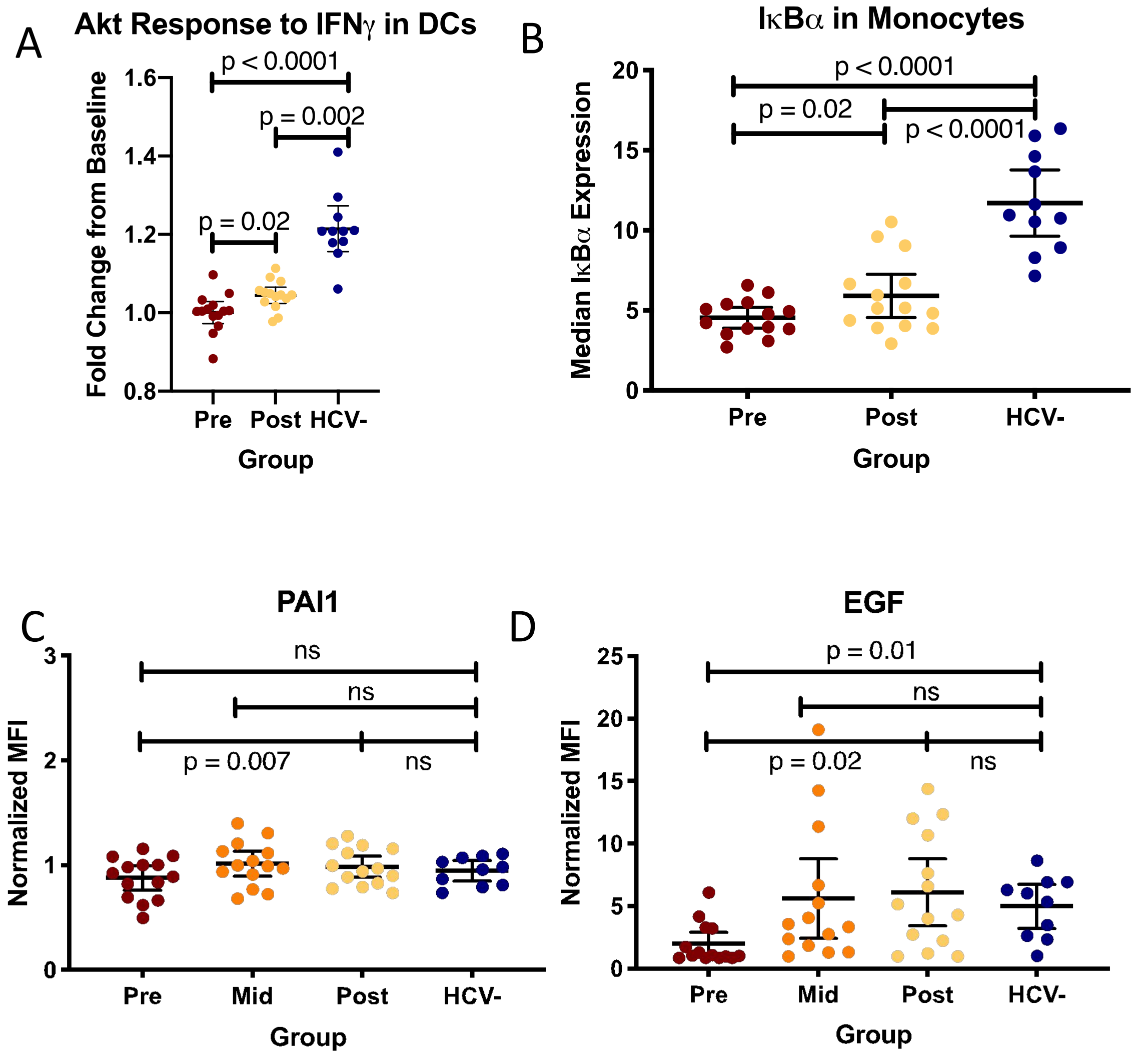


**Figure S4. Sensitivity to IFN and systemic cytokines levels altered by viral clearance.** A) Fold-changes in pAkt in DCs of the HCV cohort upon *ex-vivo* stimulation with IFNγ. Minor improvement seen with paired analysis following treatment with sofosbuvir ($p = 0.02$, Wilcoxon; $q < 0.0001$, SAM). B) Baseline IκBα expression in monocytes of the HCV cohort. Minor improvement seen with paired analysis following treatment with sofosbuvir ($p = 0.02$, Wilcoxon; $q < 0.0001$, SAM). C) Systemic PAI-1 levels are altered by viral clearance (pre- vs. post-treatment $p = 0.007$, Wilcoxon; HCV+ vs HCV-, $p > 0.99$, Kruskal–Wallis one-way analysis of variance (KW); $q < 0.0001$, paired SAM). D) Systemic EGF levels are altered by viral clearance (pre- vs. post-treatment $p = 0.02$, Wilcoxon; $q < 0.0001$, SAM; pre-treatment vs. HCV- $p = 0.01$, KW; mid- and post-treatment vs. HCV- $p > 0.99$, KW).

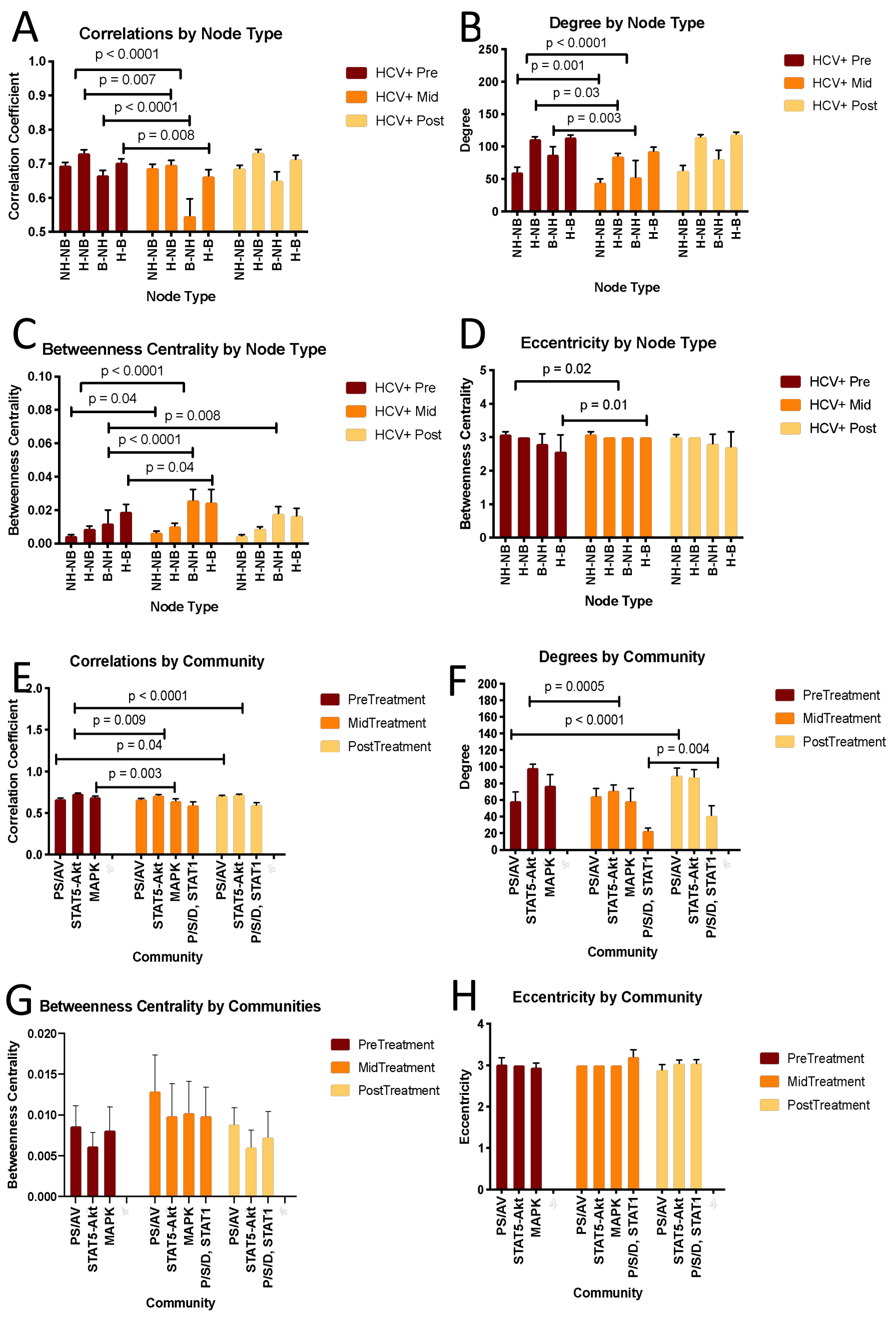


**Figure S5. Quantitative measures of immune signaling network topology throughout sofosbuvir treatment.** A) Mean Spearman correlation coefficients of node type shows that nodes of the HCV+ Pre network are more highly interconnected than the HCV+ Mid network ($p < 0.0001$) except for NH-NB nodes. B) Mean degrees by node type shows that nodes of the HCV+ Pre network interacts with more neighbors than the HCV+ Mid network ($p < 0.0001$) except fort H-B nodes. C) Mean betweenness centralities of nodes by node type demonstrates that nodes of the Pre network are the “shortest paths” in the network less frequently than the Mid network ($p < 0.0001$) except for H-NB nodes. D) Mean node eccentricity by node type demonstrates that bottleneck nodes of the Pre network are less functionally influential than those of the Mid network ($p < 0.02$). E) Mean Spearman correlation coefficients of nodes by community shows that Pre network nodes across

communities are more highly interconnected than the Mid and Post networks. F) Mean degrees of nodes by communities shows that nodes in the PS/AV and P/S/D, STAT1 communities pick up more neighbors as treatment advances, while nodes of the STAT5-Akt community drop in neighbors from Pre network to Mid network. G) Betweenness centrality and H) Eccentricity stratified by community are unchanged across networks. In all panels, mean with error bars indicating 95% confidence interval for each plot. p values for A-D were determined by two-way analysis of variance, while p values for E-H were determined paired t-test.

| HCV Cohort | | | | | | | | | |
|---|---|---|---|---|---|---|---|---|---|
| **ID** | **Previous IFN** | **Genotype** | **Treatment*** | **Liver transplant waitlist** | **Outcome** | **Sex** | **Age** | **CMV** | *Treatment Codes |
| 2 | Yes | 1 | SOF<br>SIM | No | SVR | Male | 58 | Yes | SOF = Sofosbuvir<br>SIM = Simeprevir |
| 4 | Yes | 1 | SOF<br>SIM | No | SVR | Female | 63 | Yes | RBV = Ribavirin<br>PEG = Pegylated IFN |
| 7 | No | 2 | SOF<br>RBV | No | SVR | Female | 71 | Yes | **Median(Range) |
| 8 | Yes | 2 | SOF<br>RBV | No | SVR | Female | 59 | Yes | |
| 9 | Yes | 1 | SOF<br>SIM | No | SVR | Male | 29 | Yes | |
| 12 | No | 2 | SOF<br>RBV | No | SVR | Female | 60 | No | |
| 13 | No | 1 | SOF<br>SIM | Yes | Relapse | Female | 52 | Yes | |
| 14 | Yes | 1 | SOF<br>SIM | No | SVR | Male | 65 | Yes | |
| 20 | Yes | 1 | SOF<br>SIM | No | SVR | Male | 61 | Yes | |
| 22 | Yes | 1 | SOF<br>PEG<br>RBV | No | SVR | Female | 53 | Yes | |
| 29 | Yes | 1 | SOF<br>SIM | Yes | SVR | Male | 61 | Yes | |
| 30 | No | 4 | SOF<br>PEG<br>RBV | No | SVR | Female | 57 | Yes | |
| 35 | No | 1 | SOF<br>SIM | No | SVR | Male | 53 | Yes | |
| 38 | Yes | 1 | SOF<br>SIM | No | SVR | Male | 66 | Yes | |
| | | | | | **Control Group N = 11** | F(72%) | 61(18-74)** | 64% | |

**Table S1. Clinical information for HCV cohort.**

| HIV Cohort | | | | |
|---|---|---|---|---|
| Length of Suppression (months) | Sex | Age | CMV | **Median(Range) |
| 34 | M | 26 | Yes | |
| 46 | M | 30 | Yes | |
| 13 | M | 35 | Yes | |
| 45 | M | 36 | No | |
| Unknown | M | 37 | Yes | |
| 70 | M | 38 | No | |
| Unknown | M | 38 | Yes | |
| 17 | M | 38 | Yes | |
| 86 | M | 39 | No | |
| 137 | M | 40 | Yes | |
| 19 | M | 40 | No | |
| 17 | M | 40 | Yes | |
| 13 | M | 54 | Yes | |
| 87 | M | 56 | Yes | |
| 41 | M | 56 | Yes | |
| 145 | M | 57 | Yes | |
| Unknown | M | 57 | No | |
| 69 | M | 57 | No | |
| Unknown | M | 59 | Yes | |
| 98 | M | 62 | Yes | |
| 23 | M | 64 | Yes | |
| 66 | M | 67 | No | |
| 36 | M | 70 | Yes | |
| 30 | M | 78 | Yes | |
| **Control Group N = 45** | M(100%) | 63(25-78)** | 51% | |

**Table S2. Clinical information for HIV cohort.**

| Aging Cohort | | | | |
|---|---|---|---|---|
| **Group** | **Sex** | **Age** | **CMV** | **Median(Range) |
| Young, N=29 | F(45%) | 26(22-33)** | 48% | |
| Old, N=60 | F(62%) | 79(61-90)** | 57% | |

**Table S3. Demographic information for Aging cohort.**

| Analyte | Category | Cohort |
|---|---|---|
| ENA78 | Chemokine | All |
| EOTAXIN | Chemokine | All |
| GROA | Chemokine | All |
| IL8 | Chemokine | All |
| IP10 | Chemokine | All |
| MCP1 | Chemokine | All |
| MCP3 | Chemokine | All |
| MIG | Chemokine | All |
| MIP1A | Chemokine | All |
| MIP1B | Chemokine | All |
| RANTES | Chemokine | All |
| SDF1A | Chemokine | HCV only |
| BDNF | Growth Factor | HCV only |
| VEGF | Growth Factor | All |
| PDGFBB | Growth Factor | All |
| FGFB | Growth Factor | All |
| TGFA | Growth Factor | All |
| VEGFD | Growth Factor | All |
| NGF | Growth Factor | All |
| EGF | Growth Factor | HCV only |
| HGF | Growth Factor | All |
| RESISTIN | Other | All |
| LEPTIN | Other | All |
| PAI1 | Other | All |
| ICAM1 | Other | HCV only |
| VCAM1 | Other | All |
| CD40L | Cytokine | HCV only |
| FASL | Cytokine | All |
| IL17F | Cytokine | All |
| IL27 | Cytokine | HCV only |
| LIF | Cytokine | All |
| IL1B | Cytokine | All |
| IL2 | Cytokine | All |
| IL4 | Cytokine | All |
| IL5 | Cytokine | All |
| IL6 | Cytokine | All |
| IL7 | Cytokine | All |
| IL10 | Cytokine | All |
| IFNB | Cytokine | All |
| IL12P70 | Cytokine | All |
| IL13 | Cytokine | All |
| IL17A | Cytokine | All |
| IL31 | Cytokine | HCV only |
| IL1RA | Cytokine | All |
| SCF | Cytokine | All |
| IFNG | Cytokine | All |
| GMCSF | Cytokine | All |
| TNFA | Cytokine | All |
| IFNA | Cytokine | All |
| TGFB | Cytokine | All |
| IL9 | Cytokine | HCV only |
| TNFB | Cytokine | All |
| TRAIL | Cytokine | HCV only |
| GCSF | Cytokine | All |
| IL1A | Cytokine | All |
| IL23 | Cytokine | HCV only |
| IL12P40 | Cytokine | All |
| IL15 | Cytokine | All |
| IL18 | Cytokine | All |
| MCSF | Cytokine | All |
| IL21 | Cytokine | HCV only |
| IL22 | Cytokine | HCV only |

**Table S4. Luminex cytokine panels.**

| Barcoding, L/D, DNA | Clone | Label | Supplier |
|---|---|---|---|
| CD45 | HI30 | Pd104/106/108, In115 | BD, eBioscience |
| Cisplatin | N/A | Pt195 | Enzolife |
| DNA Intercalator | N/A | Ir191/193 | Fluidigm |
| **T Cell Panel** | | | |
| CD3 | UCHT1 | Er170 | Fluidigm |
| CD4 | RPA-T4 | Nd145 | Fluidigm |
| CD8 | RPA-T8 | Nd146 | Fluidigm |
| TCRgd | 5A6.E9 | Nd142 | In House |
| CD45RA | HI100 | Sm149 | eBioscience |
| CCR7 | G043H7 | Er167 | Biolegend |
| CD28 | CD28.2 | Dy163 | BD |
| CXCR5 | RF8B2 | Yb171 | BD |
| **Activation Markers** | | | |
| CD38 | HIT2 | Cd112/114 | Lifetechnologies |
| HLA-DR | Immu357 | Dy164 | Beckman |
| CD28 | CD28.2 | Dy163 | BD |
| CD27 | LG.7F9 | Nd144 | BD |
| CD70 | 113-16 | Gd157 | Biolegend |
| **NK Panel** | | | |
| CD16 | 3G8 | Ho165 | Fluidigm |
| CD56 | HCD56 | Yb176 | Beckman |
| **B Cell Panel** | | | |
| CD20 | 2H7 | Pr141 | BD |
| CD19 | HIB19 | Nd142 | Fluidigm |
| IgD | IA6-2 | Nd145 | Biolegend |
| **Myeloid Panel** | | | |
| CD33 | WM53 | Yb173 | Biolegend |
| CD14 | M5E2 | Gd160 | Fluidigm |
| CD123 | 6H6 | Eu151 | Fluidigm |
| CD11c | 3.9 | Sm154 | Biolegend |
| **SIRPa/CD47** | | | |
| CD47 | B6H12 | La139 | BD |
| SIRPa | SE5A5 | Gd155 | BioLegend |
| **Exhaustion Markers** | | | |
| CD244 | 2B4 | Gd156 | R&D |
| PD-1 | MIH4 | Dy161 | BD |
| CD57 | HCD57 | Sm147 | Biolegend |
| CD85j | 292319 | In113 | R&D |
| **Proliferation, Survival, and** | | | |
| pCreb (pS133) | 87G3 | Nd148 | CST |
| pAkt (pS473) | D9E | Sm152 | CST |
| pS6 (pS235/S236) | N7548 | Yb172 | BD |
| **Pathogen-sensing/antiviral** | | | |
| IkBa | L35A5 | Er166 | CST |
| pIRF7 (pS477/pS479) | K47-671 | Yb174 | BD |
| **MAPK Subfamily** | | | |
| pERK1/2 (pT202/pY204) | 20A | Er168 | BD |
| pMAPKAPK2 (pT334) | 27B7 | Gd158 | CST |
| pp38 (pT180/pY182) | 36 | Tm169 | BD |
| **Cytokine Signaling** | | | |
| pSTAT1 (pY701) | 4a | Eu153 | BD |
| pSTAT3 (pY705) | 4 | Tb159 | BD |
| pSTAT4 (pY693) | D2E4 | Lu175 | CST |
| pSTAT5 (pY694) | 47 | Nd150 | BD |

**Table S5. CyTOF panel.**

| Feature | Functional Category | Data Type | Beta | Q Value |
|---|---|---|---|---|
| CD8.CD28- | Advanced T Cell Differentiation | Cell Count | 0.362 | 0.008 |
| GD.T. | Decreased gd T Frequency | Cell Count | -0.261 | 0.05 |
| cd20.BCR.pPLC | Diminished Sensitivity to BCR Stimulation | BCR Sensitivity | -0.383 | 0.008 |
| cd20.IFNa.STAT3 | Diminished Sensitivity to Cytokine in B Cells | Cytokine Sensitivity | -0.301 | 0.012 |
| cd20.IFNg.STAT3 | Diminished Sensitivity to Cytokine in B Cells | Cytokine Sensitivity | -0.226 | 0.088 |
| cd20.IL6.STAT1 | Diminished Sensitivity to Cytokine in B Cells | Cytokine Sensitivity | -0.244 | 0.082 |
| cd20.IL6.STAT3 | Diminished Sensitivity to Cytokine in B Cells | Cytokine Sensitivity | -0.305 | 0.02 |
| cd20.IL7.STAT3 | Diminished Sensitivity to Cytokine in B Cells | Cytokine Sensitivity | -0.23 | 0.086 |
| cd4.IFNa.STAT1 | Diminished Sensitivity to Cytokine in CD4T | Cytokine Sensitivity | -0.207 | 0.164 |
| cd4.IFNa.STAT5 | Diminished Sensitivity to Cytokine in CD4T | Cytokine Sensitivity | -0.246 | 0.063 |
| cd4.IL21.STAT1 | Diminished Sensitivity to Cytokine in CD4T | Cytokine Sensitivity | -0.296 | 0.04 |
| cd4.IL21.STAT5 | Diminished Sensitivity to Cytokine in CD4T | Cytokine Sensitivity | -0.235 | 0.109 |
| cd4.IL6.STAT1 | Diminished Sensitivity to Cytokine in CD4T | Cytokine Sensitivity | -0.267 | 0.042 |
| cd4.IL6.STAT5 | Diminished Sensitivity to Cytokine in CD4T | Cytokine Sensitivity | -0.507 | 0.008 |
| cd8.IFNa.STAT1 | Diminished Sensitivity to Cytokine in CD8T | Cytokine Sensitivity | -0.508 | 0.008 |
| cd8.IFNa.STAT3 | Diminished Sensitivity to Cytokine in CD8T | Cytokine Sensitivity | -0.207 | 0.15 |
| cd8.IFNa.STAT5 | Diminished Sensitivity to Cytokine in CD8T | Cytokine Sensitivity | -0.452 | 0.008 |
| cd8.IFNg.STAT1 | Diminished Sensitivity to Cytokine in CD8T | Cytokine Sensitivity | -0.643 | 0.008 |
| cd8.IFNg.STAT5 | Diminished Sensitivity to Cytokine in CD8T | Cytokine Sensitivity | -0.217 | 0.123 |
| cd8.IL21.STAT1 | Diminished Sensitivity to Cytokine in CD8T | Cytokine Sensitivity | -0.441 | 0.008 |
| cd8.IL21.STAT5 | Diminished Sensitivity to Cytokine in CD8T | Cytokine Sensitivity | -0.266 | 0.064 |
| cd8.IL6.STAT1 | Diminished Sensitivity to Cytokine in CD8T | Cytokine Sensitivity | -0.599 | 0.008 |
| cd8.IL6.STAT3 | Diminished Sensitivity to Cytokine in CD8T | Cytokine Sensitivity | -0.407 | 0.008 |
| cd8.IL6.STAT5 | Diminished Sensitivity to Cytokine in CD8T | Cytokine Sensitivity | -0.625 | 0.008 |
| cd8.IL7.STAT1 | Diminished Sensitivity to Cytokine in CD8T | Cytokine Sensitivity | -0.409 | 0.008 |
| mono.IFNa.STAT3 | Diminished Sensitivity to Cytokine in Monos | Cytokine Sensitivity | -0.276 | 0.038 |
| mono.IFNg.STAT3 | Diminished Sensitivity to Cytokine in Monos | Cytokine Sensitivity | -0.321 | 0.007 |
| mono.IL10.STAT3 | Diminished Sensitivity to Cytokine in Monos | Cytokine Sensitivity | -0.264 | 0.064 |
| mono.IL2.STAT3 | Diminished Sensitivity to Cytokine in Monos | Cytokine Sensitivity | -0.195 | 0.184 |
| mono.IL6.STAT3 | Diminished Sensitivity to Cytokine in Monos | Cytokine Sensitivity | -0.307 | 0.016 |
| cd20.U.STAT3 | Elevated Baseline Signaling in B Cells | Baseline Signaling | 0.239 | 0.077 |
| cd4.U.STAT1 | Elevated Baseline Signaling in CD4T | Baseline Signaling | 0.327 | 0.016 |
| cd4.U.STAT5 | Elevated Baseline Signaling in CD4T | Baseline Signaling | 0.25 | 0.068 |
| cd8.U.STAT1 | Elevated Baseline Signaling in CD8T | Baseline Signaling | 0.572 | 0.008 |
| mono.U.STAT3 | Elevated Baseline Signaling in Monos | Baseline Signaling | 0.213 | 0.13 |
| IL-12P40 | Elevated IL12p40 | Cytokine Level | 0.321 | 0.012 |
| IL-12-P70 | Elevated IL12p70 | Cytokine Level | 0.259 | 0.052 |
| IL-18 | Elevated IL18 | Cytokine Level | 0.221 | 0.15 |
| IL-1b | Elevated IL1B | Cytokine Level | 0.205 | 0.164 |
| IL-8 | Elevated IL8 | Cytokine Level | 0.224 | 0.109 |
| MIP-1B | Elevated MIP1B | Cytokine Level | 0.2 | 0.164 |
| SCF | Elevated SCF | Cytokine Level | 0.393 | 0.008 |
| TNF-a | Elevated TNFA | Cytokine Level | 0.218 | 0.164 |
| TNF-b | Elevated TNFB | Cytokine Level | 0.25 | 0.04 |
| cd4.IL10.STAT5 | Enhanced Sensitivity to Cytokine in CD4T | Cytokine Sensitivity | 0.207 | 0.164 |
| cd4.IL2.STAT1 | Enhanced Sensitivity to Cytokine in CD4T | Cytokine Sensitivity | 0.205 | 0.15 |
| cd4.IL2.STAT3 | Enhanced Sensitivity to Cytokine in CD4T | Cytokine Sensitivity | 0.238 | 0.116 |
| cd4.IL2.STAT5 | Enhanced Sensitivity to Cytokine in CD4T | Cytokine Sensitivity | 0.264 | 0.042 |
| cd8.IL10.STAT3 | Enhanced Sensitivity to Cytokine in CD8T | Cytokine Sensitivity | 0.277 | 0.048 |
| cd8.IL10.STAT5 | Enhanced Sensitivity to Cytokine in CD8T | Cytokine Sensitivity | 0.268 | 0.052 |
| cd8.IL2.STAT3 | Enhanced Sensitivity to Cytokine in CD8T | Cytokine Sensitivity | 0.256 | 0.064 |
| cd8.IL2.STAT5 | Enhanced Sensitivity to Cytokine in CD8T | Cytokine Sensitivity | 0.296 | 0.02 |
| Monos | Increased Monocyte Counts | Cell Count | 0.32 | 0.012 |
| NK. | Increased NK Counts | Cell Count | 0.446 | 0.008 |
| CD4.CM | T Cell Memory Skewing | Cell Count | 0.396 | 0.008 |
| CD4.Naive | T Cell Memory Skewing | Cell Count | -0.432 | 0.008 |
| CD8.CM | T Cell Memory Skewing | Cell Count | 0.41 | 0.012 |
| CD8.EM | T Cell Memory Skewing | Cell Count | 0.227 | 0.071 |
| CD8.Naive | T Cell Memory Skewing | Cell Count | -0.603 | 0.008 |
| CD8.TEM | T Cell Memory Skewing | Cell Count | 0.249 | 0.077 |

**Table S6. Immune features significantly altered by aging.**

| Feature | Functional Category | Data Type | Beta | Q Value |
|---|---|---|---|---|
| EGF | Decreased EGF | Cytokine Level | -0.71369864 | 0.062 |
| %gdT_in_ActivatedT | Decreased gd T Frequency | Cell Count | -0.42236757 | 0.11621212 |
| pp38_B | Depressed Baseline MAPK Signaling in B Cells | Baseline Signaling | -0.59697562 | 0.01934426 |
| pErk1.2_Monos | Depressed Baseline MAPK Signaling in Monos | Baseline Signaling | -0.42627529 | 0.13080189 |
| pp38_Monos | Depressed Baseline MAPK Signaling in Monos | Baseline Signaling | -0.53963173 | 0.04326667 |
| pErk1.2_NKs | Depressed Baseline MAPK Signaling in NKs | Baseline Signaling | -0.40532817 | 0.15945946 |
| pp38_NKs | Depressed Baseline MAPK Signaling in NKs | Baseline Signaling | -0.54302757 | 0.04326667 |
| pSTAT4_Monos | Depressed Baseline STAT4 Signaling in Monos | Baseline Signaling | -0.71093868 | 0.00842857 |
| IFNa_B_pAkt | Diminished Sensitivity to Cytokine in B Cells | Cytokine Sensitivity | -0.57938691 | 0.02723077 |
| IFNa_B_pERK1_2 | Diminished Sensitivity to Cytokine in B Cells | Cytokine Sensitivity | -0.47756879 | 0.01525862 |
| IFNa_B_pP38 | Diminished Sensitivity to Cytokine in B Cells | Cytokine Sensitivity | -0.77990714 | 0.00842857 |
| IFNa_B_pSTAT4 | Diminished Sensitivity to Cytokine in B Cells | Cytokine Sensitivity | -0.54820942 | 0.05756098 |
| IFNg_B_pAkt | Diminished Sensitivity to Cytokine in B Cells | Cytokine Sensitivity | -0.65870425 | 0.00842857 |
| IFNg_B_pCreb | Diminished Sensitivity to Cytokine in B Cells | Cytokine Sensitivity | -0.48216957 | 0.07531915 |
| IFNg_B_pERK1_2 | Diminished Sensitivity to Cytokine in B Cells | Cytokine Sensitivity | -0.59911726 | 0.01156863 |
| IFNg_B_pIRF7 | Diminished Sensitivity to Cytokine in B Cells | Cytokine Sensitivity | -0.51514449 | 0.06247059 |
| IFNg_B_pMAPKAPK2 | Diminished Sensitivity to Cytokine in B Cells | Cytokine Sensitivity | -0.40384763 | 0.19666667 |
| IFNg_B_pP38 | Diminished Sensitivity to Cytokine in B Cells | Cytokine Sensitivity | -0.87856482 | 0.00842857 |
| IFNg_B_pSTAT1 | Diminished Sensitivity to Cytokine in B Cells | Cytokine Sensitivity | -0.50783232 | 0.06807692 |
| IFNg_B_pSTAT3 | Diminished Sensitivity to Cytokine in B Cells | Cytokine Sensitivity | -0.63257201 | 0.01525862 |
| IFNg_B_pSTAT4 | Diminished Sensitivity to Cytokine in B Cells | Cytokine Sensitivity | -0.864163 | 0.00842857 |
| IFNg_B_pSTAT5 | Diminished Sensitivity to Cytokine in B Cells | Cytokine Sensitivity | -0.72827094 | 0.00842857 |
| IFNa_CD4T_pAkt | Diminished Sensitivity to Cytokine in CD4T | Cytokine Sensitivity | -0.49745392 | 0.06555556 |
| IFNa_CD4T_pERK1_2 | Diminished Sensitivity to Cytokine in CD4T | Cytokine Sensitivity | -0.45495153 | 0.01934426 |
| IFNa_CD4T_pP38 | Diminished Sensitivity to Cytokine in CD4T | Cytokine Sensitivity | -0.74261192 | 0.00842857 |
| IFNg_CD4T_pAkt | Diminished Sensitivity to Cytokine in CD4T | Cytokine Sensitivity | -0.49833025 | 0.0479375 |
| IFNg_CD4T_pERK1_2 | Diminished Sensitivity to Cytokine in CD4T | Cytokine Sensitivity | -0.63570568 | 0.00686047 |
| IFNg_CD4T_pIRF7 | Diminished Sensitivity to Cytokine in CD4T | Cytokine Sensitivity | -0.46065268 | 0.11683168 |
| IFNg_CD4T_pP38 | Diminished Sensitivity to Cytokine in CD4T | Cytokine Sensitivity | -0.83834518 | 0.00842857 |
| IFNg_CD4T_pSTAT3 | Diminished Sensitivity to Cytokine in CD4T | Cytokine Sensitivity | -0.72429193 | 0.00686047 |
| IFNg_CD4T_pSTAT4 | Diminished Sensitivity to Cytokine in CD4T | Cytokine Sensitivity | -0.77284012 | 0.00842857 |
| IFNg_CD4T_pSTAT5 | Diminished Sensitivity to Cytokine in CD4T | Cytokine Sensitivity | -0.58936849 | 0.02723077 |
| IFNa_CD8T_pAkt | Diminished Sensitivity to Cytokine in CD8T | Cytokine Sensitivity | -0.48487655 | 0.07295699 |
| IFNa_CD8T_pERK1_2 | Diminished Sensitivity to Cytokine in CD8T | Cytokine Sensitivity | -0.48145316 | 0.01156863 |
| IFNa_CD8T_pP38 | Diminished Sensitivity to Cytokine in CD8T | Cytokine Sensitivity | -0.69244065 | 0.00842857 |
| IFNg_CD8T_pAkt | Diminished Sensitivity to Cytokine in CD8T | Cytokine Sensitivity | -0.54580648 | 0.04326667 |
| IFNg_CD8T_pERK1_2 | Diminished Sensitivity to Cytokine in CD8T | Cytokine Sensitivity | -0.66304988 | 0.01156863 |
| IFNg_CD8T_pIRF7 | Diminished Sensitivity to Cytokine in CD8T | Cytokine Sensitivity | -0.41867926 | 0.18274336 |
| IFNg_CD8T_pP38 | Diminished Sensitivity to Cytokine in CD8T | Cytokine Sensitivity | -0.87942067 | 0.00842857 |
| IFNg_CD8T_pSTAT3 | Diminished Sensitivity to Cytokine in CD8T | Cytokine Sensitivity | -0.76041997 | 0.00842857 |
| IFNg_CD8T_pSTAT4 | Diminished Sensitivity to Cytokine in CD8T | Cytokine Sensitivity | -0.77764946 | 0.00842857 |
| IFNa_DCs_pAkt | Diminished Sensitivity to Cytokine in DCs | Cytokine Sensitivity | -0.63710478 | 0.01156863 |
| IFNa_DCs_pIRF7 | Diminished Sensitivity to Cytokine in DCs | Cytokine Sensitivity | -0.44231446 | 0.13080189 |
| IFNa_DCs_pP38 | Diminished Sensitivity to Cytokine in DCs | Cytokine Sensitivity | -0.49436722 | 0.05970238 |
| IFNg_DCs_pAkt | Diminished Sensitivity to Cytokine in DCs | Cytokine Sensitivity | -0.89362571 | 0.00842857 |
| IFNg_DCs_pCreb | Diminished Sensitivity to Cytokine in DCs | Cytokine Sensitivity | -0.52877524 | 0.0415493 |
| IFNg_DCs_pERK1_2 | Diminished Sensitivity to Cytokine in DCs | Cytokine Sensitivity | -0.59068639 | 0.01525862 |
| IFNg_DCs_pIRF7 | Diminished Sensitivity to Cytokine in DCs | Cytokine Sensitivity | -0.49954503 | 0.06369318 |
| IFNg_DCs_pMAPKAPK2 | Diminished Sensitivity to Cytokine in DCs | Cytokine Sensitivity | -0.4543173 | 0.11137755 |
| IFNg_DCs_pP38 | Diminished Sensitivity to Cytokine in DCs | Cytokine Sensitivity | -0.73331626 | 0.00842857 |
| IFNg_DCs_pSTAT1 | Diminished Sensitivity to Cytokine in DCs | Cytokine Sensitivity | -0.65079244 | 0.01934426 |
| IFNg_DCs_pSTAT4 | Diminished Sensitivity to Cytokine in DCs | Cytokine Sensitivity | -0.67138438 | 0.00686047 |
| IFNg_DCs_pSTAT5 | Diminished Sensitivity to Cytokine in DCs | Cytokine Sensitivity | -0.61548299 | 0.01525862 |
| IFNa_gdT_pERK1_2 | Diminished Sensitivity to Cytokine in gdT | Cytokine Sensitivity | -0.4010221 | 0.13261468 |
| IFNg_gdT_pERK1_2 | Diminished Sensitivity to Cytokine in gdT | Cytokine Sensitivity | -0.87663357 | 0.00842857 |
| IFNg_gdT_pP38 | Diminished Sensitivity to Cytokine in gdT | Cytokine Sensitivity | -0.94277444 | 0.00842857 |
| IFNg_gdT_pSTAT3 | Diminished Sensitivity to Cytokine in gdT | Cytokine Sensitivity | -0.62670002 | 0.01525862 |
| IFNg_gdT_pSTAT4 | Diminished Sensitivity to Cytokine in gdT | Cytokine Sensitivity | -0.82460256 | 0.00842857 |
| IFNg_Monos_pAkt | Diminished Sensitivity to Cytokine in Monos | Cytokine Sensitivity | -0.6987968 | 0.00842857 |
| IFNg_Monos_pCreb | Diminished Sensitivity to Cytokine in Monos | Cytokine Sensitivity | -0.69674108 | 0.00842857 |
| IFNg_Monos_pERK1_2 | Diminished Sensitivity to Cytokine in Monos | Cytokine Sensitivity | -0.52431703 | 0.04597403 |
| IFNg_Monos_pIRF7 | Diminished Sensitivity to Cytokine in Monos | Cytokine Sensitivity | -0.55756845 | 0.0479375 |
| IFNg_Monos_pMAPKAPK2 | Diminished Sensitivity to Cytokine in Monos | Cytokine Sensitivity | -0.58650852 | 0.01525862 |
| IFNg_Monos_pP38 | Diminished Sensitivity to Cytokine in Monos | Cytokine Sensitivity | -0.72914518 | 0.00842857 |
| IFNa_NK_pERK1_2 | Diminished Sensitivity to Cytokine in NKs | Cytokine Sensitivity | -0.38490238 | 0.15945946 |
| IFNg_NK_pAkt | Diminished Sensitivity to Cytokine in NKs | Cytokine Sensitivity | -0.61838043 | 0.00842857 |
| IFNg_NK_pERK1_2 | Diminished Sensitivity to Cytokine in NKs | Cytokine Sensitivity | -0.63326434 | 0.01525862 |
| IFNg_NK_pIRF7 | Diminished Sensitivity to Cytokine in NKs | Cytokine Sensitivity | -0.38741461 | 0.19666667 |
| IFNg_NK_pP38 | Diminished Sensitivity to Cytokine in NKs | Cytokine Sensitivity | -0.85387622 | 0.00842857 |
| IFNg_NK_pSTAT4 | Diminished Sensitivity to Cytokine in NKs | Cytokine Sensitivity | -0.72755318 | 0.00686047 |
| IFNg_DCs_pSTAT3 | Diminished Sensitivity to Cytokine in DCs | Cytokine Sensitivity | -0.43665683 | 0.13233645 |
| %CD4T_in_CD28LoT | Diminished T Cell Differentiation | Cell Count | -0.51133753 | 0.07763158 |
| pAkt_B | Elevated Baseline Signaling in B Cells | Baseline Signaling | 0.461946116 | 0.11857843 |
| pSTAT1_B | Elevated Baseline Signaling in B Cells | Baseline Signaling | 0.735274955 | 0.00842857 |
| pSTAT3_B | Elevated Baseline Signaling in B Cells | Baseline Signaling | 0.417626286 | 0.18274336 |
| pSTAT5_B | Elevated Baseline Signaling in B Cells | Baseline Signaling | 0.669630678 | 0.01156863 |
| pAkt_CD4T | Elevated Baseline Signaling in CD4T | Baseline Signaling | 0.548702246 | 0.05970238 |
| pCreb_CD4T | Elevated Baseline Signaling in CD4T | Baseline Signaling | 0.531347449 | 0.03904412 |
| pS6_CD4T | Elevated Baseline Signaling in CD4T | Baseline Signaling | 0.504432621 | 0.06369318 |
| pSTAT1_CD4T | Elevated Baseline Signaling in CD4T | Baseline Signaling | 0.734508818 | 0.00842857 |
| pSTAT3_CD4T | Elevated Baseline Signaling in CD4T | Baseline Signaling | 0.705275029 | 0.00842857 |
| pSTAT4_CD4T | Elevated Baseline Signaling in CD4T | Baseline Signaling | 0.441063076 | 0.12764423 |
| pSTAT5_CD4T | Elevated Baseline Signaling in CD4T | Baseline Signaling | 0.745343475 | 0.00842857 |
| pAkt_CD8T | Elevated Baseline Signaling in CD8T | Baseline Signaling | 0.487837363 | 0.09526042 |
| pS6_CD8T | Elevated Baseline Signaling in CD8T | Baseline Signaling | 0.420748023 | 0.13261468 |
| pSTAT1_CD8T | Elevated Baseline Signaling in CD8T | Baseline Signaling | 0.651650735 | 0.00842857 |
| pSTAT3_CD8T | Elevated Baseline Signaling in CD8T | Baseline Signaling | 0.665973644 | 0.00842857 |
| pSTAT5_CD8T | Elevated Baseline Signaling in CD8T | Baseline Signaling | 0.700967727 | 0.00686047 |
| IkBa_DCs | Elevated Baseline Signaling in DCs | Baseline Signaling | -0.48827428 | 0.07054348 |
| pAkt_DCs | Elevated Baseline Signaling in DCs | Baseline Signaling | 0.533263627 | 0.06369318 |
| pSTAT1_DCs | Elevated Baseline Signaling in DCs | Baseline Signaling | 0.495635625 | 0.04326667 |
| pSTAT3_DCs | Elevated Baseline Signaling in DCs | Baseline Signaling | 0.66068446 | 0.01156863 |
| pSTAT5_DCs | Elevated Baseline Signaling in DCs | Baseline Signaling | 0.724895967 | 0.00686047 |
| IkBa_gdT | Elevated Baseline Signaling in gdT | Baseline Signaling | -0.54373578 | 0.03904412 |
| pSTAT1_gdT | Elevated Baseline Signaling in gdT | Baseline Signaling | 0.677803239 | 0.00842857 |
| pSTAT5_gdT | Elevated Baseline Signaling in gdT | Baseline Signaling | 0.567542724 | 0.0415493 |
| IkBa_Monos | Elevated Baseline Signaling in Monos | Baseline Signaling | -0.78834199 | 0.00842857 |
| pAkt_Monos | Elevated Baseline Signaling in Monos | Baseline Signaling | 0.39288401 | 0.19666667 |
| pS6_Monos | Elevated Baseline Signaling in Monos | Baseline Signaling | 0.39725436 | 0.19666667 |
| pSTAT1_Monos | Elevated Baseline Signaling in Monos | Baseline Signaling | 0.530238117 | 0.03575758 |
| pSTAT5_Monos | Elevated Baseline Signaling in Monos | Baseline Signaling | 0.656246286 | 0.00686047 |
| pSTAT1_NKs | Elevated Baseline Signaling in NKs | Baseline Signaling | 0.420507911 | 0.12601942 |
| pSTAT5_NKs | Elevated Baseline Signaling in NKs | Baseline Signaling | 0.46048504 | 0.10036083 |
| IL12P40 | Elevated IL12p40 | Cytokine Level | 0.562558841 | 0.1988 |
| IL1RA | Elevated IL1RA | Cytokine Level | 0.493050572 | 0.15525 |
| IP10 | Elevated IP10 | Cytokine Level | 0.516893605 | 0.166 |
| RANTES | Elevated RANTES | Cytokine Level | 0.524493667 | 0.16885714 |
| RESISTIN | Elevated RESISTIN | Cytokine Level | 0.633670761 | 0.094 |
| TNFA | Elevated TNFA | Cytokine Level | 0.527762942 | 0.1435 |
| VCAM1 | Elevated VCAM1 | Cytokine Level | 0.552344647 | 0.17566667 |
| VEGF | Elevated VEGF | Cytokine Level | 0.477468323 | 0.1741 |
| VEGFD | Elevated VEGFD | Cytokine Level | 0.625890855 | 0.12533333 |
| IFNg_B_IkBa | Enhanced Sensitivity to Cytokine in B Cells | Cytokine Sensitivity | -0.68227961 | 0.00686047 |
| IFNg_B_pS6 | Enhanced Sensitivity to Cytokine in B Cells | Cytokine Sensitivity | 0.553341032 | 0.0415493 |
| IFNg_CD4T_IkBa | Enhanced Sensitivity to Cytokine in CD4T | Cytokine Sensitivity | -0.71176892 | 0.00842857 |
| IFNg_CD4T_pS6 | Enhanced Sensitivity to Cytokine in CD4T | Cytokine Sensitivity | 0.55482201 | 0.02723077 |
| IFNg_CD8T_IkBa | Enhanced Sensitivity to Cytokine in CD8T | Cytokine Sensitivity | -0.50932962 | 0.0479375 |
| IFNg_CD8T_pS6 | Enhanced Sensitivity to Cytokine in CD8T | Cytokine Sensitivity | 0.698788606 | 0.00842857 |
| IFNg_DCs_pS6 | Enhanced Sensitivity to Cytokine in DCs | Cytokine Sensitivity | 0.394497819 | 0.19666667 |
| IFNg_gdT_IkBa | Enhanced Sensitivity to Cytokine in gdT | Cytokine Sensitivity | -0.53490511 | 0.04597403 |
| IFNa_gdT_pMAPKAPK2 | Enhanced Sensitivity to Cytokine in gdT | Cytokine Sensitivity | 0.480788238 | 0.06555556 |
| IFNa_gdT_pS6 | Enhanced Sensitivity to Cytokine in gdT | Cytokine Sensitivity | 0.533546578 | 0.05756098 |
| IFNg_gdT_pS6 | Enhanced Sensitivity to Cytokine in gdT | Cytokine Sensitivity | 0.579255416 | 0.01156863 |
| IFNg_Monos_pS6 | Enhanced Sensitivity to Cytokine in Monos | Cytokine Sensitivity | 0.776034427 | 0.00842857 |
| IFNa_Monos_pS6 | Enhanced Sensitivity to Cytokine in Monos | Cytokine Sensitivity | 0.389663007 | 0.19666667 |
| IFNa_Monos_pSTAT5 | Enhanced Sensitivity to Cytokine in Monos | Cytokine Sensitivity | 0.443113096 | 0.11683168 |
| IFNg_NK_IkBa | Enhanced Sensitivity to Cytokine in NKs | Cytokine Sensitivity | -0.6243296 | 0.01156863 |
| IFNg_NK_pS6 | Enhanced Sensitivity to Cytokine in NKs | Cytokine Sensitivity | 0.687024366 | 0.00842857 |
| IFNa_NK_pSTAT5 | Enhanced Sensitivity to Cytokine in NKs | Cytokine Sensitivity | 0.395989885 | 0.19666667 |
| %CD4T_in_NaiveCD4T | T Cell Memory Skewing | Cell Count | -0.55421935 | 0.02379032 |

**Table S7. Immune features significantly altered by HCV infection.**

| Feature | Functional Category | Data Type | Beta | Q Value |
|---|---|---|---|---|
| CD4 | Decreased CD4 Count | Cell Count | -0.61416422 | 0.16 |
| cd4_cd8ratio | Decreased CD4 Count | Cell Count | -0.40942425 | 0.128 |
| cd20.IFNa.STAT3 | Diminished Sensitivity to Cytokine in B Cells | Cytokine Sensitivity | -0.41254789 | 0.1993472 |
| cd4.IFNa.STAT1 | Diminished Sensitivity to Cytokine in CD4T | Cytokine Sensitivity | -0.51681127 | 0.1993472 |
| cd4.IFNa.STAT5 | Diminished Sensitivity to Cytokine in CD4T | Cytokine Sensitivity | -0.40491284 | 0.19991284 |
| cd4.IL6.STAT5 | Diminished Sensitivity to Cytokine in CD4T | Cytokine Sensitivity | -0.65334671 | 0.17066667 |
| cd8.IFNa.STAT3 | Diminished Sensitivity to Cytokine in CD8T | Cytokine Sensitivity | -0.55116868 | 0.1792 |
| cd8.IL6.STAT3 | Diminished Sensitivity to Cytokine in CD8T | Cytokine Sensitivity | -0.47851292 | 0.1992517 |
| cd8.IL7.STAT5 | Diminished Sensitivity to Cytokine in CD8T | Cytokine Sensitivity | -0.42863865 | 0.18773333 |
| mono.IFNa.STAT3 | Diminished Sensitivity to Cytokine in Monos | Cytokine Sensitivity | -0.51123238 | 0.18773333 |
| mono.IFNa.STAT5 | Diminished Sensitivity to Cytokine in Monos | Cytokine Sensitivity | -0.47658007 | 0.192 |
| CD8.CD28- | Diminished T Cell Differentiation | Cell Count | -0.57811963 | 0.064 |
| cd20.Unstimulated.STAT3 | Elevated Baseline Signaling in B Cells | Baseline Signaling | 0.410708822 | 0.18773333 |
| cd20.Unstimulated.STAT5 | Elevated Baseline Signaling in B Cells | Baseline Signaling | 0.431654922 | 0.18488889 |
| cd4.Unstimulated.STAT1 | Elevated Baseline Signaling in CD4T | Baseline Signaling | 0.455432009 | 0.19781818 |
| cd4.Unstimulated.STAT5 | Elevated Baseline Signaling in CD4T | Baseline Signaling | 0.484476329 | 0.18863158 |
| cd8.Unstimulated.STAT1 | Elevated Baseline Signaling in CD8T | Baseline Signaling | 0.513366389 | 0.10971429 |
| cd8.Unstimulated.STAT5 | Elevated Baseline Signaling in CD8T | Baseline Signaling | 0.425133057 | 0.1995392 |
| mono.Unstimulated.STAT5 | Elevated Baseline Signaling in Monos | Baseline Signaling | 0.526882252 | 0.128 |
| CD4.EM | T Cell Memory Skewing | Cell Count | 0.3352 | 0.19781818 |
| CD4.Naive | T Cell Memory Skewing | Cell Count | -0.49310968 | 0.18618182 |
| cd4na_memRatio | T Cell Memory Skewing | Cell Count | -0.3080387 | 0.1024 |
| CD8.EM | T Cell Memory Skewing | Cell Count | 0.238 | 0.18488889 |
| CD8.Naive | T Cell Memory Skewing | Cell Count | -0.50936989 | 0.10971429 |
| cd8na_memRatio | T Cell Memory Skewing | Cell Count | -0.5698413 | 0.064 |

**Table S8. Immune features significantly altered by HIV infection.**

| Immune Parameter | FDR | Direction | Data Type |
|---|---|---|---|
| IkBa_Monos | <1% | Increased | Baseline Signaling |
| PAI1 | <1% | Increased | Cytokine Level |
| EGF | <1% | Increased | Cytokine Level |
| IFNa_CD4T_pSTAT1 | <1% | Increased | Cytokine Sensitivity |
| IFNa_CD8T_pSTAT1 | <1% | Increased | Cytokine Sensitivity |
| IFNg_DCs_pAkt | <1% | Increased | Cytokine Sensitivity |
| IFNa_B_pSTAT1 | <1% | Increased | Cytokine Sensitivity |
| IFNa_gdT_pSTAT1 | <1% | Increased | Cytokine Sensitivity |
| IFNa_DCs_pSTAT3 | <1% | Increased | Cytokine Sensitivity |
| IFNa_NK_pSTAT1 | <1% | Increased | Cytokine Sensitivity |
| IFNa_DCs_pSTAT5 | <1% | Increased | Cytokine Sensitivity |
| IFNa_Monos_pSTAT1 | <1% | Increased | Cytokine Sensitivity |
| IFNa_DCs_pSTAT1 | <1% | Increased | Cytokine Sensitivity |

**Table S9. Immune parameters altered by viral clearance in the HCV cohort.**

| Nodes | Pre-Treatment Degree | Pre-Treatment Betweenness Centrality | Pre-Treatment Community | Mid-Treatment Degree | Mid-Treatment Betweenness Centrality | Mid-Treatment Community | Post-Treatment Degree | Post-Treatment Betweenness Centrality | Post-Treatment Community |
|---|---|---|---|---|---|---|---|---|---|
| IkBa_Bcells | 44 | 0.00203906 | PS/AV | 20 | 0.00927948 | P/S/D, STAT1 | 90 | 0.00633427 | PS/AV |
| IkBa_CD4T | 42 | 0.02659812 | PS/AV | 24 | 0.01245514 | P/S/D, STAT1 | 24 | 0.00646165 | PS/AV |
| IkBa_CD8T | 28 | 0.000336 | PS/AV | 20 | 0.00873204 | P/S/D, STAT1 | 6 | 0.000142 | P/S/D, STAT1 |
| IkBa_DCs | 20 | 0.000986 | PS/AV | 30 | 0.02849709 | STAT5-Akt | 4 | 0 | PS/AV |
| IkBa_gdT | 52 | 0.00226963 | PS/AV | 22 | 0.00527149 | PS/AV | 56 | 0.00358935 | PS/AV |
| IkBa_Monos | 34 | 0.000611 | PS/AV | 10 | 0.00226085 | P/S/D, STAT1 | 54 | 0.03158573 | PS/AV |
| IkBa_NKs | 100 | 0.0041734 | STAT5-Akt | 50 | 0.00273715 | STAT5-Akt | 90 | 0.00670387 | STAT5-Akt |
| pAkt_Bcells | 84 | 0.00292385 | STAT5-Akt | 82 | 0.00896801 | STAT5-Akt | 90 | 0.00533607 | STAT5-Akt |
| pAkt_CD4T | 84 | 0.00148796 | STAT5-Akt | 72 | 0.00305439 | STAT5-Akt | 68 | 0.00167573 | STAT5-Akt |
| pAkt_CD8T | 90 | 0.00216545 | STAT5-Akt | 78 | 0.00359143 | STAT5-Akt | 78 | 0.00113921 | STAT5-Akt |
| pAkt_DCs | 102 | 0.01057299 | STAT5-Akt | 68 | 0.00522755 | STAT5-Akt | 96 | 0.00312939 | STAT5-Akt |
| pAkt_gdT | 80 | 0.00120981 | STAT5-Akt | 70 | 0.00249118 | STAT5-Akt | 76 | 0.00238024 | STAT5-Akt |
| pAkt_Monos | 98 | 0.00302727 | STAT5-Akt | 76 | 0.00318824 | STAT5-Akt | 104 | 0.00457059 | STAT5-Akt |
| pAkt_NKs | 100 | 0.00323985 | STAT5-Akt | 78 | 0.003749 | STAT5-Akt | 100 | 0.0039131 | STAT5-Akt |
| pCreb_Bcells | 20 | 0.00200572 | PS/AV | 18 | 0.00418715 | P/S/D, STAT1 | 20 | 0.00158881 | P/S/D, STAT1 |
| pCreb_CD4T | 20 | 0.00399639 | PS/AV | 36 | 0.02664536 | P/S/D, STAT1 | 28 | 0.00791065 | P/S/D, STAT1 |
| pCreb_CD8T | 46 | 0.01321609 | PS/AV | 34 | 0.0207731 | P/S/D, STAT1 | 34 | 0.00688748 | P/S/D, STAT1 |
| pCreb_DCs | 102 | 0.014317 | PS/AV | 46 | 0.00890643 | PS/AV | 78 | 0.01262326 | P/S/D, STAT1 |
| pCreb_gdT | 94 | 0.00873862 | PS/AV | 66 | 0.01808299 | PS/AV | 84 | 0.00947948 | STAT5-Akt |
| pCreb_Monos | 56 | 0.00937563 | PS/AV | 12 | 0.00101258 | P/S/D, STAT1 | 18 | 0.000191 | P/S/D, STAT1 |
| pCreb_NKs | 110 | 0.01542684 | PS/AV | 92 | 0.0224018 | PS/AV | 114 | 0.01290038 | STAT5-Akt |
| pErk1.2_Bcells | 44 | 0.000786 | MAPK | 32 | 0.00101294 | MAPK | 48 | 0.000138 | PS/AV |
| pErk1.2_CD4T | 20 | 0 | MAPK | 26 | 0.0039017 | MAPK | 40 | 0 | PS/AV |
| pErk1.2_CD8T | 48 | 0.00493889 | MAPK | 60 | 0.0048078 | MAPK | 80 | 0.00374544 | PS/AV |
| pErk1.2_DCs | 76 | 0.00439047 | MAPK | 88 | 0.01283272 | MAPK | 96 | 0.00403253 | PS/AV |
| pErk1.2_gdT | 50 | 0.00250153 | MAPK | 64 | 0.0084776 | MAPK | 82 | 0.00522207 | PS/AV |
| pErk1.2_Monos | 68 | 0.00240681 | MAPK | 72 | 0.00634863 | MAPK | 86 | 0.01840385 | PS/AV |
| pErk1.2_NKs | 78 | 0.00486157 | MAPK | 78 | 0.01919745 | MAPK | 84 | 0.00196725 | PS/AV |
| pIRF7_Bcells | 116 | 0.02177783 | MAPK | 64 | 0.00625716 | PS/AV | 90 | 0.00689071 | PS/AV |
| pIRF7_CD4T | 88 | 0.01858106 | MAPK | 60 | 0.00375663 | PS/AV | 104 | 0.01296796 | PS/AV |
| pIRF7_CD8T | 98 | 0.01518821 | PS/AV | 62 | 0.00548062 | PS/AV | 102 | 0.01221372 | PS/AV |
| pIRF7_DCs | 64 | 0.01020092 | PS/AV | 30 | 0.00797796 | PS/AV | 74 | 0.01553072 | P/S/D, STAT1 |
| pIRF7_gdT | 116 | 0.02578148 | PS/AV | 64 | 0.00605592 | PS/AV | 114 | 0.0197681 | PS/AV |
| pIRF7_Monos | 100 | 0.01524704 | PS/AV | 64 | 0.0415591 | PS/AV | 90 | 0.00966611 | PS/AV |
| pIRF7_NKs | 114 | 0.02383436 | PS/AV | 64 | 0.00795334 | PS/AV | 120 | 0.01052141 | PS/AV |
| pMAPKAPK2_Bcells | 84 | 0.01894044 | PS/AV | 18 | 0.00263096 | P/S/D, STAT1 | 30 | 0.00626332 | P/S/D, STAT1 |
| pMAPKAPK2_CD4T | 62 | 0.01343497 | PS/AV | 28 | 0.01340397 | P/S/D, STAT1 | 24 | 0.00322241 | P/S/D, STAT1 |
| pMAPKAPK2_CD8T | 94 | 0.01445488 | PS/AV | 30 | 0.00932879 | PS/AV | 68 | 0.01757487 | P/S/D, STAT1 |
| pMAPKAPK2_DCs | 80 | 0.00586758 | PS/AV | 50 | 0.01035357 | PS/AV | 100 | 0.00963737 | PS/AV |
| pMAPKAPK2_gdT | 114 | 0.01432401 | PS/AV | 82 | 0.03399114 | PS/AV | 100 | 0.01128598 | STAT5-Akt |
| pMAPKAPK2_Monos | 56 | 0.00542667 | PS/AV | 28 | 0.01336466 | P/S/D, STAT1 | 34 | 0.00397482 | P/S/D, STAT1 |
| pMAPKAPK2_NKs | 110 | 0.00891436 | PS/AV | 76 | 0.00871486 | STAT5-Akt | 120 | 0.01513805 | STAT5-Akt |
| pp38_Bcells | 108 | 0.00871676 | STAT5-Akt | 90 | 0.01870852 | STAT5-Akt | 122 | 0.01207314 | PS/AV |
| pp38_CD4T | 100 | 0.01148138 | MAPK | 56 | 0.03214568 | STAT5-Akt | 90 | 0.00414028 | STAT5-Akt |
| pp38_CD8T | 114 | 0.00897418 | STAT5-Akt | 88 | 0.00820717 | STAT5-Akt | 110 | 0.00617756 | STAT5-Akt |
| pp38_DCs | 120 | 0.01760884 | MAPK | 66 | 0.01167567 | MAPK | 92 | 0.00268542 | PS/AV |
| pp38_gdT | 120 | 0.01279495 | MAPK | 98 | 0.01174392 | STAT5-Akt | 116 | 0.00904372 | STAT5-Akt |
| pp38_Monos | 38 | 0.000953 | MAPK | 28 | 0.00531145 | MAPK | 26 | 0.002216 | P/S/D, STAT1 |
| pp38_NKs | 110 | 0.00719859 | STAT5-Akt | 66 | 0.01483208 | MAPK | 108 | 0.00592453 | PS/AV |
| pS6_Bcells | 20 | 0.00140528 | PS/AV | 32 | 0.01542065 | P/S/D, STAT1 | 20 | 0.00167141 | P/S/D, STAT1 |
| pS6_CD4T | 20 | 0.00243092 | PS/AV | 30 | 0.01158912 | P/S/D, STAT1 | 32 | 0.0090875 | P/S/D, STAT1 |
| pS6_CD8T | 34 | 0.00663624 | PS/AV | 26 | 0.00709395 | P/S/D, STAT1 | 28 | 0.00588001 | P/S/D, STAT1 |
| pS6_DCs | 76 | 0.00483522 | PS/AV | 66 | 0.00939793 | PS/AV | 32 | 0.000781 | P/S/D, STAT1 |
| pS6_gdT | 48 | 0.00209433 | PS/AV | 30 | 0.00398934 | P/S/D, STAT1 | 38 | 0.0022389 | STAT5-Akt |
| pS6_Monos | 84 | 0.0054603 | PS/AV | 52 | 0.00263722 | PS/AV | 30 | 0.00064 | P/S/D, STAT1 |
| pS6_NKs | 52 | 0.00791955 | PS/AV | 30 | 0.00718495 | P/S/D, STAT1 | 54 | 0.00941529 | STAT5-Akt |
| pSTAT1_Bcells | 16 | 0.000311 | PS/AV | 30 | 0.01714663 | MAPK | 106 | 0.00727579 | PS/AV |
| pSTAT1_CD4T | 28 | 0.00514226 | PS/AV | 16 | 0.00255657 | P/S/D, STAT1 | 82 | 0.00564367 | P/S/D, STAT1 |
| pSTAT1_CD8T | 34 | 0.00254318 | PS/AV | 20 | 0.00333832 | P/S/D, STAT1 | 54 | 0.02459389 | P/S/D, STAT1 |
| pSTAT1_DCs | 22 | 0.00408684 | PS/AV | 18 | 0.00431712 | P/S/D, STAT1 | 20 | 0.00126974 | P/S/D, STAT1 |
| pSTAT1_gdT | 32 | 0.00218564 | PS/AV | 24 | 0.01362499 | P/S/D, STAT1 | 58 | 0.00817559 | P/S/D, STAT1 |
| pSTAT1_Monos | 30 | 0.00220108 | PS/AV | 22 | 0.00487797 | P/S/D, STAT1 | 38 | 0.00506297 | P/S/D, STAT1 |
| pSTAT1_NKs | 88 | 0.02686422 | PS/AV | 20 | 0.00817885 | P/S/D, STAT1 | 124 | 0.0186054 | STAT5-Akt |
| pSTAT3_Bcells | 78 | 0.00925122 | MAPK | 106 | 0.03784373 | STAT5-Akt | 112 | 0.0074488 | PS/AV |
| pSTAT3_CD4T | 4 | 0 | PS/AV | 12 | 0.00428323 | P/S/D, STAT1 | 68 | 0.00492529 | PS/AV |
| pSTAT3_CD8T | 30 | 0.00803241 | MAPK | 30 | 0.03836263 | P/S/D, STAT1 | 100 | 0.00688247 | PS/AV |
| pSTAT3_DCs | 104 | 0.00595847 | MAPK | 82 | 0.01045769 | STAT5-Akt | 120 | 0.00959665 | PS/AV |
| pSTAT3_gdT | 80 | 0.01540943 | MAPK | 94 | 0.01746474 | MAPK | 120 | 0.01104253 | PS/AV |
| pSTAT3_Monos | 82 | 0.00605702 | MAPK | 22 | 0.00712874 | P/S/D, STAT1 | 60 | 0.00647798 | PS/AV |
| pSTAT3_NKs | 90 | 0.00442795 | MAPK | 90 | 0.01462678 | PS/AV | 116 | 0.00957065 | PS/AV |
| pSTAT4_Bcells | 100 | 0.00394648 | STAT5-Akt | 74 | 0.00251205 | STAT5-Akt | 102 | 0.01052806 | PS/AV |
| pSTAT4_CD4T | 84 | 0.00412436 | MAPK | 76 | 0.00837103 | PS/AV | 98 | 0.0045429 | PS/AV |
| pSTAT4_CD8T | 116 | 0.01061851 | STAT5-Akt | 80 | 0.00963557 | PS/AV | 94 | 0.00889255 | PS/AV |
| pSTAT4_DCs | 116 | 0.01114517 | STAT5-Akt | 84 | 0.00612063 | STAT5-Akt | 118 | 0.01034802 | PS/AV |
| pSTAT4_gdT | 106 | 0.00644842 | STAT5-Akt | 92 | 0.01622995 | PS/AV | 104 | 0.01856287 | PS/AV |
| pSTAT4_Monos | 110 | 0.01367362 | MAPK | 94 | 0.02596953 | PS/AV | 82 | 0.01474246 | PS/AV |
| pSTAT4_NKs | 98 | 0.01715056 | STAT5-Akt | 80 | 0.00979056 | PS/AV | 118 | 0.01132619 | PS/AV |
| pSTAT5_Bcells | 96 | 0.00268754 | STAT5-Akt | 64 | 0.00373361 | STAT5-Akt | 70 | 0.000517 | STAT5-Akt |
| pSTAT5_CD4T | 84 | 0.00595835 | STAT5-Akt | 58 | 0.00582493 | STAT5-Akt | 70 | 0.00128832 | STAT5-Akt |
| pSTAT5_CD8T | 96 | 0.0082419 | STAT5-Akt | 64 | 0.00699548 | STAT5-Akt | 68 | 0.001119 | STAT5-Akt |
| pSTAT5_DCs | 98 | 0.00781248 | STAT5-Akt | 60 | 0.00655786 | STAT5-Akt | 92 | 0.01102667 | STAT5-Akt |
| pSTAT5_gdT | 90 | 0.00415208 | STAT5-Akt | 50 | 0.01160154 | STAT5-Akt | 72 | 0.00125952 | STAT5-Akt |
| pSTAT5_Monos | 84 | 0.00142093 | STAT5-Akt | 80 | 0.01164947 | STAT5-Akt | 118 | 0.02539045 | P/S/D, STAT1 |
| pSTAT5_NKs | 110 | 0.00813134 | STAT5-Akt | 52 | 0.00203031 | STAT5-Akt | 84 | 0.00258171 | STAT5-Akt |

**Table S10. Node characteristics of pre-, mid-, and post-treatment networks of HCV cohort.**

| Immune Signaling Correlation Network | | | |
|---|---|---|---|
| **Pre-Treatment** | | | |
| Hub Enrichment | Category | Fold Enrichment | P Value (by hypergeometric test) |
| | p38 | 3.53 | 0.003 |
| | STAT4 | 2.82 | 0.03 |
| Bottleneck Enrichment | IRF7 | 4.24 | 0.0002 |
| **Mid-Treatment** | | | |
| Hub Enrichment | Category | Fold Enrichment | P Value (by hypergeometric test) |
| | STAT3 | 2.82 | 0.03 |
| | STAT4 | 3.53 | 0.003 |
| **Post-Treatment** | | | |
| Hub Enrichment | Category | Fold Enrichment | P Value (by hypergeometric test) |
| | NKs | 2.88 | 0.002 |
| | p38 | 2.82 | 0.03 |
| | STAT3 | 2.82 | 0.03 |
| Hub and Bottleneck(H-B) Enrichment | NKs | 2.2 | 0.05 |
| Bottleneck Enrichment | IRF7 | 2.82 | 0.03 |
| | | | |
| **Modularity Analysis by Communities** | | | |
| **Pre-Treatment** | | | |
| Pink Module Enrichment | Category | Fold Enrichment | P Value (by hypergeometric test) |
| | Pathogen Sensing/Antiviral | 1.65 | 0.01 |
| | Proliferation/Survival/Differentiation | 1.4 | 0.04 |
| | STAT1 | 2.1 | 0.004 |
| Green Module Enrichment | STAT5-AKT | 3.65 | 2.57 x 10^-10 |
| | STAT4 | 2.61 | 0.02 |
| Black Module Enrichment | MAPK | 2.1 | 0.002 |
| | pSTAT3 | 3.43 | 0.0008 |
| **Mid-Treatment** | | | |
| Pink Module Enrichment | Category | Fold Enrichment | P Value (by hypergeometric test) |
| | Pathogen Sensing/Antiviral | 2.18 | 0.007 |
| | STAT4 | 2.73 | 0.01 |
| Green Module Enrichment | STAT5-AKT | 3.36 | 1.4 x 10^-9 |
| Black Module Enrichment | MAPK | 3.33 | 6.3 x 10^-6 |
| Blue Module Enrichment | STAT1 | 2.88 | 0.002 |
| | pS6 | 2.4 | 0.02 |
| **Post-Treatment** | | | |
| Pink Module Enrichment | Category | Fold Enrichment | P Value (by hypergeometric test) |
| | Pathogen Sensing/Antiviral | 1.78 | 0.005 |
| | STAT3,4 and ERK1/2 | 2.27 | 0.002 |
| Green Module Enrichment | STAT5-AKT | 3.25 | 4.8 x 10^-8 |
| Blue Module Enrichment | Proliferation/Survival/Differentiation | 1.74 | 0.02 |
| | pSTAT1 | 2.62 | 0.02 |

**Table S11. Network and community enrichment analysis.**

| Node | Pre vs. Post p value | Mid vs. Post p value | Pre vs. Mid p value |
|---|---|---|---|
| IkBa_Bcells | 0.0677 | 0.3879 | 0.3647 |
| IkBa_CD4T | 0.0591 | 0 | 0.3897 |
| IkBa_CD8T | 0.0819 | 0.0935 | 0.1922 |
| IkBa_DCs | 0.0485 | 0.0339 | 0.3792 |
| IkBa_gdT | 0.0119 | 0.1311 | 0.4692 |
| IkBa_Monos | 0.0519 | 0.1812 | 0.6603 |
| IkBa_NKs | 0.0359 | 0.18 | 0.5814 |
| pAkt_Bcells | 0.0125 | 0.0368 | 0.1102 |
| pAkt_CD4T | 0.0466 | 0.0356 | 0.1008 |
| pAkt_CD8T | 0.0346 | 0.0106 | 0.2057 |
| pAkt_DCs | 0.0349 | 0.0937 | 0.4774 |
| pAkt_gdT | 0 | 0.074 | 0.2202 |
| pAkt_Monos | 0.0122 | 0.2403 | 0.5429 |
| pAkt_NKs | 0.0105 | 0.0829 | 0.3877 |
| pCreb_Bcells | 0.0612 | 0.0821 | 0.1407 |
| pCreb_CD4T | 0.0443 | 0.0679 | 0.0766 |
| pCreb_CD8T | 0.0469 | 0.0136 | 0.1429 |
| pCreb_DCs | 0.0457 | 0.1098 | 0.5881 |
| pCreb_gdT | 0.0121 | 0.0888 | 0.327 |
| pCreb_Monos | 0.072 | 0.039 | 0.626 |
| pCreb_NKs | 0 | 0.066 | 0.2389 |
| pErk1.2_Bcells | 0.0121 | 0.0216 | 0.0369 |
| pErk1.2_CD4T | 0.0501 | 0.0383 | 0.0577 |
| pErk1.2_CD8T | 0.0481 | 0.0693 | 0.0739 |
| pErk1.2_DCs | 0.045 | 0.0379 | 0.1663 |
| pErk1.2_gdT | 0.051 | 0.082 | 0.0509 |
| pErk1.2_Monos | 0.0241 | 0.0812 | 0.1562 |
| pErk1.2_NKs | 0.0118 | 0.0363 | 0.1109 |
| pIRF7_Bcells | 0.0371 | 0.1821 | 0.5567 |
| pIRF7_CD4T | 0.0365 | 0.2623 | 0.4971 |
| pIRF7_CD8T | 0.051 | 0.2692 | 0.4806 |
| pIRF7_DCs | 0 | 0.2273 | 0.5512 |
| pIRF7_gdT | 0.0121 | 0.2235 | 0.614 |
| pIRF7_Monos | 0.0116 | 0.1323 | 0.4227 |
| pIRF7_NKs | 0 | 0.2253 | 0.5054 |
| pMAPKAPK2_Bcells | 0.0501 | 0.1659 | 0.707 |
| pMAPKAPK2_CD4T | 0.1067 | 0.2313 | 0.8621 |
| pMAPKAPK2_CD8T | 0.0224 | 0.0676 | 0.3475 |
| pMAPKAPK2_DCs | 0.045 | 0.2401 | 0.3826 |
| pMAPKAPK2_gdT | 0.0486 | 0.0702 | 0.5104 |
| pMAPKAPK2_Monos | 0.0617 | 0.0212 | 0.4352 |
| pMAPKAPK2_NKs | 0.0102 | 0.1802 | 0.4911 |
| pp38_Bcells | 0.046 | 0.1789 | 0.2537 |
| pp38_CD4T | 0.0238 | 0.1276 | 0.4821 |
| pp38_CD8T | 0 | 0.1339 | 0.4117 |
| pp38_DCs | 0.049 | 0.3286 | 0.8446 |
| pp38_gdT | 0.0222 | 0.118 | 0.4709 |
| pp38_Monos | 0.0253 | 0.0683 | 0.364 |
| pp38_NKs | 0.0118 | 0.2668 | 0.67 |
| pS6_Bcells | 0.1077 | 0.0348 | 0.7214 |
| pS6_CD4T | 0.0605 | 0.0471 | 0.1719 |
| pS6_CD8T | 0.0118 | 0.0803 | 0.2177 |
| pS6_DCs | 0.0643 | 0.1095 | 0.084 |
| pS6_gdT | 0.0526 | 0.0342 | 0.3517 |
| pS6_Monos | 0.1062 | 0.0265 | 0.5544 |
| pS6_NKs | 0.0098 | 0.1289 | 0.3113 |
| pSTAT1_Bcells | 0.2546 | 0.4469 | 0.0252 |
| pSTAT1_CD4T | 0.1062 | 0.667 | 0.4522 |
| pSTAT1_CD8T | 0.0506 | 0.2271 | 0.3096 |
| pSTAT1_DCs | 0.0347 | 0.1013 | 0.1548 |
| pSTAT1_gdT | 0.0469 | 0.2232 | 0.3208 |
| pSTAT1_Monos | 0.0352 | 0.136 | 0.1931 |
| pSTAT1_NKs | 0.0453 | 0.8092 | 0.8177 |
| pSTAT3_Bcells | 0.0592 | 0.0349 | 0.2484 |
| pSTAT3_CD4T | 0.3695 | 0.3455 | 0.4558 |
| pSTAT3_CD8T | 0.2125 | 0.2788 | 0.2039 |
| pSTAT3_DCs | 0.0087 | 0.0916 | 0.2796 |
| pSTAT3_gdT | 0.0813 | 0.0856 | 0.2162 |
| pSTAT3_Monos | 0.025 | 0.1534 | 0.5184 |
| pSTAT3_NKs | 0.0464 | 0.0696 | 0.0303 |
| pSTAT4_Bcells | 0.0114 | 0.1424 | 0.4735 |
| pSTAT4_CD4T | 0.0473 | 0.1199 | 0.1129 |
| pSTAT4_CD8T | 0 | 0.0815 | 0.2945 |
| pSTAT4_DCs | 0.0259 | 0.2031 | 0.4404 |
| pSTAT4_gdT | 0 | 0.011 | 0.0059 |
| pSTAT4_Monos | 0.0624 | 0.0756 | 0.1875 |
| pSTAT4_NKs | 0.0346 | 0.1421 | 0.2365 |
| pSTAT5_Bcells | 0.072 | 0.0378 | 0.5614 |
| pSTAT5_CD4T | 0.0423 | 0.0819 | 0.4861 |
| pSTAT5_CD8T | 0.0454 | 0.0724 | 0.471 |
| pSTAT5_DCs | 0.0246 | 0.1794 | 0.59 |
| pSTAT5_gdT | 0.047 | 0.1203 | 0.622 |
| pSTAT5_Monos | 0.0484 | 0.0827 | 0.0186 |
| pSTAT5_NKs | 0.0721 | 0.1766 | 0.777 |

**Table S12. Network robustness analysis comparing differences in observed networks to null networks.**
